# Molecular Architecture of the Mouse Nervous System

**DOI:** 10.1016/j.cell.2018.06.021

**Published:** 2018-08-09

**Authors:** Amit Zeisel, Hannah Hochgerner, Peter Lönnerberg, Anna Johnsson, Fatima Memic, Job van der Zwan, Martin Häring, Emelie Braun, Lars E. Borm, Gioele La Manno, Simone Codeluppi, Alessandro Furlan, Kawai Lee, Nathan Skene, Kenneth D. Harris, Jens Hjerling-Leffler, Ernest Arenas, Patrik Ernfors, Ulrika Marklund, Sten Linnarsson

**Affiliations:** 1 Division of Molecular Neurobiology, Department of Medical Biochemistry and Biophysics, Karolinska Institute, S-17177 Stockholm, Sweden; 2 UCL Institute of Neurology, London WC1N 3BG, UK

**Keywords:** RNA sequencing, cell type, single-cell transcriptomics, transcriptomics, classification

## Abstract

The mammalian nervous system executes complex behaviors controlled by specialized, precisely positioned, and interacting cell types. Here, we used RNA sequencing of half a million single cells to create a detailed census of cell types in the mouse nervous system. We mapped cell types spatially and derived a hierarchical, data-driven taxonomy. Neurons were the most diverse and were grouped by developmental anatomical units and by the expression of neurotransmitters and neuropeptides. Neuronal diversity was driven by genes encoding cell identity, synaptic connectivity, neurotransmission, and membrane conductance. We discovered seven distinct, regionally restricted astrocyte types that obeyed developmental boundaries and correlated with the spatial distribution of key glutamate and glycine neurotransmitters. In contrast, oligodendrocytes showed a loss of regional identity followed by a secondary diversification. The resource presented here lays a solid foundation for understanding the molecular architecture of the mammalian nervous system and enables genetic manipulation of specific cell types.

## Highlights

• Systematic survey of transcriptomic cell types in the mouse nervous system
• Taxonomy and hierarchical organization of molecular cell types
• Seven distinct astrocyte types with regionally restricted distribution
• Neuronal diversity is similar across brain regions

## Graphical Abstract

**Figure:**
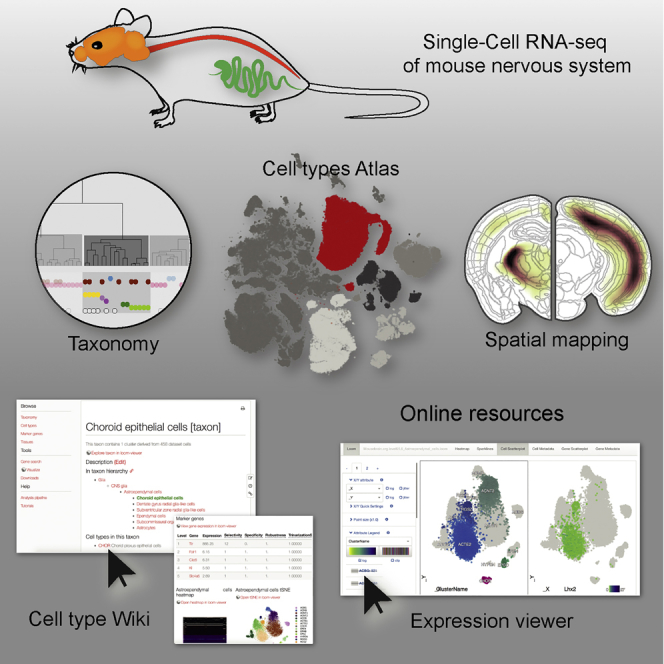

## Teaser

Single-cell transcriptional profiling of the adult mouse nervous system uncovers new cell classes and types across regions, providing a clearer picture of cell diversity by region and a reference atlas for studying the mammalian nervous system.

## Introduction

The organization of the adult mammalian nervous system is the result of developmental, functional, evolutionary, and biomechanical constraints. Our current understanding of its architecture originated with the pioneering studies of Santiago Ramón y Cajal, who mapped microscopic neuroanatomy in exquisite detail. The adult brain is organized into dorsoventral and rostrocaudal compartments, which result from patterning of the early neural tube (Rubenstein and Rakic, 2013). However, many neurons (e.g., telencephalic interneurons), glia (e.g., oligodendrocyte precursor cells [OPCs]), and vascular and immune cells migrate long distances during embryogenesis and thus end up in a location different from their place of birth. Furthermore, convergent functional specialization occurs in many parts of the nervous system: for example, dopaminergic neurons are found both in the midbrain and in the olfactory bulb, and noradrenergic neurons are found in the sympathetic ganglia, as well as the hindbrain.

The question therefore arises as to whether the molecular identity of a cell is determined mainly by its developmental ancestry, by its local environment, or by its function. All three possibilities are plausible *a priori*: neurons with shared function (for example, long-range projecting neurons or neurons using a common principal neurotransmitter) might be expected show common gene expression states across brain regions. Alternatively, chemical cues arising from a local environment might impose constraints forcing neighboring cells of different functions to become molecularly similar. Finally, developmental origin, through shared gene regulatory circuits, might retain an imprint on cell types in the adult so that gene expression patterns would reflect developmental domains and borders.

Pioneering work has revealed the broad patterns of gene expression across the mammalian brain (Lein et al., 2007). More recently, single-cell RNA sequencing (scRNA-seq) has emerged as a powerful method for unbiased discovery of cell types and states based on gene activity (Islam et al., 2011, Jaitin et al., 2014, Macosko et al., 2015, Ramsköld et al., 2012, Shekhar et al., 2016, Tang et al., 2009, Tasic et al., 2016, Usoskin et al., 2015, Zeisel et al., 2015), and initiatives are underway to create atlases of both human and model organisms (Regev et al., 2017). Here, we used systematic scRNA-seq to survey cells across the central nervous system (CNS) and peripheral nervous system (PNS). We use the inferred molecular relationships between all cell types to propose a data-driven taxonomy of cell types, and we discuss the overall architecture of the mammalian nervous system in light of this taxonomy.

## Results

### A Molecular Survey of the Mouse Nervous System

**Figure 1:**
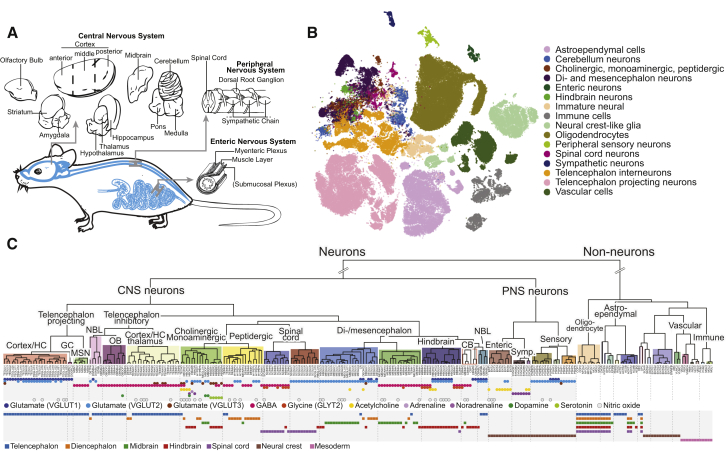
Molecular Survey of the Mouse Nervous System Using Single-Cell RNA Sequencing (A) Schematic illustration of the sampling strategy. The brain was divided into coarse anatomical units, and in addition, we sampled from the spinal cord, dorsal root ganglia, sympathetic ganglion, and enteric nervous system. (B) Visualization of the single-cell data using gt-SNE embedding (see STAR Methods). Cells are colored by rank 3 taxonomy units indicated in the legend. (C) Dendrogram describing the taxonomy of all identified cell types. Main branches, corresponding to the taxonomy, are annotated with labels and colored background. The neurotransmitter used by each cell type is indicated below the leaves as colored circles. The lower panel indicates the developmental compartment of origin for each cell types. See also Figure S1 and Tables S1, S2, S3, S4, and S5.

We performed a comprehensive survey of the adolescent mouse nervous system by scRNA-seq. We dissected the brain and spinal cord into contiguous anatomical regions and further included the peripheral sensory, enteric, and sympathetic nervous system. In total, we analyzed 19 regions (Figure 1A) but omitted at least the retina, the olfactory epithelium, the vomeronasal organ, the inner ear, and the parasympathetic ganglia.

We sampled cells without selection, except in the intestine, where we isolated neural-crest-derived cells (enteric nervous system) by FACS in *Wnt1-Cre;R26Tomato* transgenic mice. In the hippocampus and cortex we obtained additional inhibitory neurons by fluorescence-activated cell sorting (FACS) from the *vGat-Cre;TdTomato* strain. We used at least two mice for each tissue, typically one male and one female, and analyzed a total of 133 samples (Table S1) by droplet microfluidics (10X Genomics Chromium) to reveal the transcriptomes of 509,876 cells.

Preliminary analyses showed that the dataset contained hundreds of distinct cell types and that the dynamic range of cell-type abundances spanned four orders of magnitude. In addition, the dataset was affected by a number of technical artifacts, including low-quality cells, batch effects, sex-specific gene expression, neuronal-activity-dependent gene expression, and more. To overcome these challenges, we developed a multistage analysis pipeline called “cytograph,” which progressively discovers cell types or states while mitigating the impact of technical artifacts (see STAR Methods).

**Figure S1:**
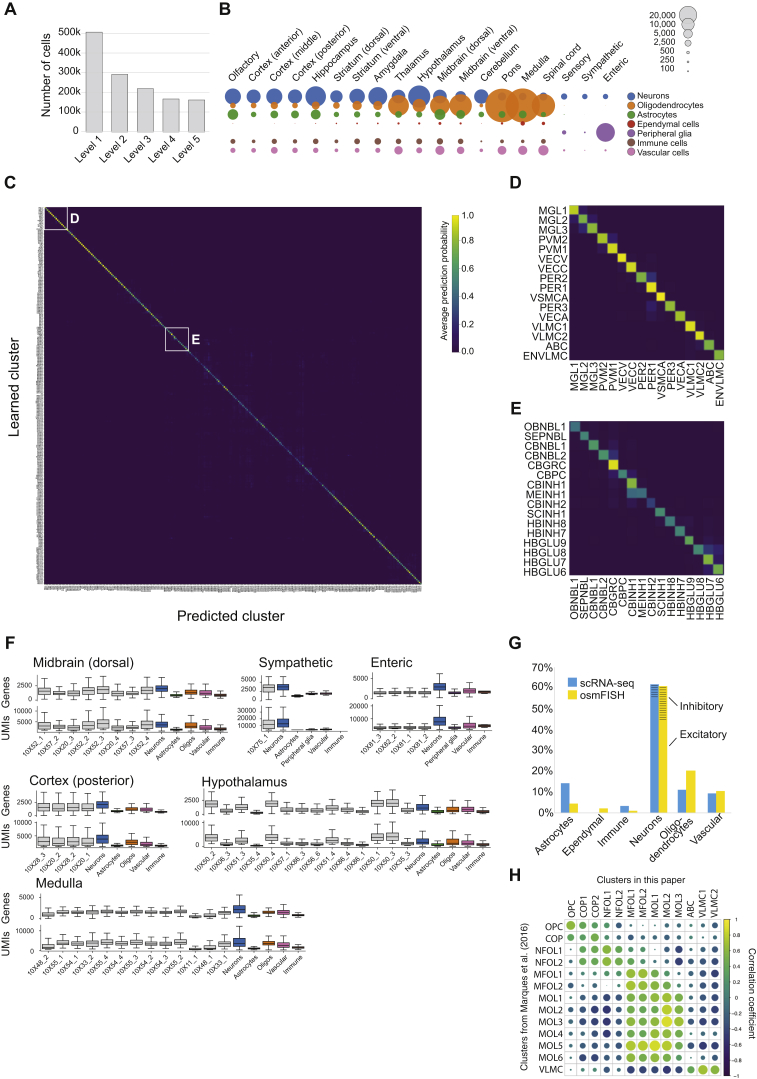
Data Quality, Related to Figure 1 (A) Number of cells retained in analysis for each level of the pipeline. (B) Circle plots showing number of cells from each main class and each dissection region. (C) Cluster robustness and relatedness. The heatmap illustrates the performance of a random forest classifier, showing the average probability assigned to every cell type (rows) for each test cell of given type (columns). When the correct cell type (diagonal) has high probability, almost every test cell will be correctly classified. (D,E) Magnified view of heatmap as indicated in (C). (F) Distribution of Gene and UMI counts for individual Chromium samples (gray) and major cell classes (colored), shown for each of a representative selection of tissues. (G) Comparison of cell type fractions observed by osmFISH (single-molecule fluorescent *in situ* hybridization) and scRNA-seq. (H) Comparison of oligodendrocyte lineage clustering in the present paper and those previously published in Marques et al., 2016.

After an initial quality assessment of samples and cells, we retained 492,949 cells as inputs to the computational analysis. During three stages of manifold learning and clustering, we removed additional doublets, outliers, and low-quality cells (Figure S1A). As oligodendrocytes are extremely abundant in the hindbrain and spinal cord, we removed more than 200,000 oligodendrocytes from these regions in order to better balance the number of oligodendrocytes between tissues (but analyzing the full set of cells did not reveal any additional structure in the oligodendrocyte lineage). The final, high-quality curated compendium comprised 265 clusters represented by 160,796 high-quality single-cell transcriptomes (Figures 1B and 1C). This represents a highly conservative clustering, and significant heterogeneity likely remains within many of the reported clusters.

To assess the robustness of the clusters, we trained a random forest classifier to recognize cluster labels and then assessed its performance on held-out data (80% training set, 20% test set). The average precision and recall were both 82%, indicating a high level of robustness, particularly considering the large total number of clusters (Figures S1C–S1E).

In order to validate our ability to recover known cell types, we assessed the concordance with six previously published and experimentally validated scRNA-seq datasets, comprising two different technologies (Fludigm C1 and 10X Genomics Chromium) and five tissues: cortex (Zeisel et al., 2015), striatum (Muñoz-Manchado et al., 2018), dentate gyrus (Hochgerner et al., 2018), spinal cord (Häring et al., 2018), and sympathetic nervous system (Furlan et al., 2016). Of the 139 previously published clusters, 98% were perfect or near-perfect matches to corresponding clusters in the new compendium (84% perfect, 14% near perfect, 2% mismatches; see Table S2).

We performed a comprehensive annotation of the clusters using a variety of automated and manual methods. We assigned each cluster a unique mnemonic identifier (e.g., MBDOP1), descriptive name (“midbrain dopaminergic neuron”), major class (e.g., “neuron”), neurotransmitter identity, putative developmental origin, anatomical location, and region (Table S3). We computed enriched genes for each cluster (indicating increased but not unique expression), as well as a probabilistic “trinarization” score, which can be used to determine if a gene is expressed, not expressed, or ambiguous in each cluster (see STAR Methods). We combined enrichment and trinarization scores to discover marker gene sets sufficient to uniquely identify each cluster with high probability (Table S4). Remarkably, we found that 248 (93%) of all clusters were uniquely identifiable with just two genes, while 17 required three genes and none required more than three (although adding more genes increased the robustness of identification).

We trained a support-vector machine classifier to automatically assign each cell to one of seven major classes: neurons, oligodendrocytes (all ∼236,000), astrocytes, ependymal cells, peripheral glia (e.g., Schwann cells, satellite, and enteric glia), immune cells, and vascular cells (Figure S1B). Neurons were most prevalent in rostral regions of the CNS, as well as in the cerebellum. In caudal regions, oligodendrocytes—needed to support long-range neurotransmission—dominated greatly, comprising 84% of cells in the hindbrain (excepting cerebellum) and 71% in the spinal cord. Astrocytes ranged from 13% of cells in the telencephalon to 6% in the hindbrain. Due to sources of bias, such as differential survival or cell capture, these estimates can only be approximate. As further validation, we compared with estimates obtained by single-molecule mRNA fluorescence *in situ* hybridization (osmFISH; Codeluppi et al., 2018) from the somatosensory cortex (Figure S1G). Notably, interneurons were undersampled by scRNA-seq, likely due to their fragility, whereas astrocytes were undersampled by osmFISH due to the difficulty of image segmentation for irregularly shaped cells with small somata.

To begin to understand the molecular organization of the mammalian nervous system, we calculated a robust dendrogram of cell types (Figure 1C and STAR Methods), showing relationships between cell types based on gene expression distance. The resulting arrangement of cell types revealed that the mammalian nervous system is organized according to three overlapping and interacting principles: major class (e.g., neurons, astrocytes), developmental origin (e.g., telencephalon, diencephalon, midbrain, hindbrain) and neurotransmitter type (e.g., GABA, glutamate).

At the top level, neurons were separated from non-neuronal types regardless of tissue, reflecting a split between major classes of cells that express thousands of genes differentially. Notably, this first split does not correspond to any shared developmental or anatomical origins, as it groups neurons from both the CNS and PNS on one side and the corresponding central glia (e.g., astrocytes) and peripheral glia (e.g., Schwann cells) on the other, along with developmentally unrelated vascular and immune cells.

PNS neurons segregated from the CNS, reflecting the developmental split between neural-crest-derived (PNS) and neural tube-derived (CNS) neurons. The peripheral neurons then split into sensory, sympathetic, and enteric subdivisions, corresponding to functional, anatomical, and developmental differences between the three major divisions of the PNS. CNS neurons generally split first by anteroposterior domain (olfactory, telencephalon, diencephalon, midbrain, hindbrain, spinal cord), and then by excitatory versus inhibitory neurotransmitter. Based on these and similar observations, we propose a data-driven molecular taxonomy arranged in a hierarchy of more than 70 named taxa (Table S5), respecting the dendrogram of Figure 1C. The taxonomy provides an objective structuring principle for exploring the global architecture of the mammalian nervous system.

### Postnatal Neurogenesis in the Central Nervous System

**Figure 2:**
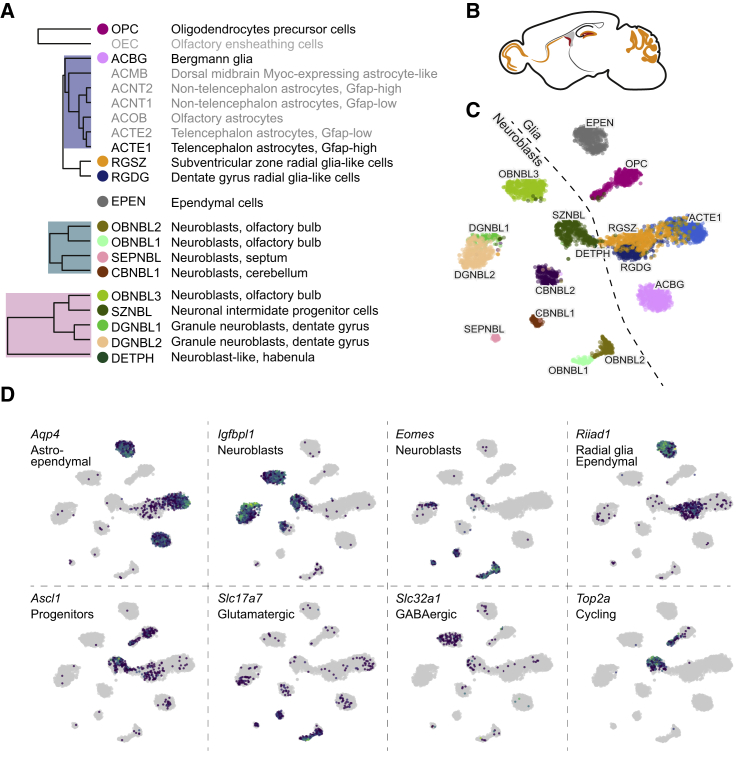
A Map of Neurogenesis in the Juvenile Mouse Brain (A) A cut-out from dendrogram of relevant cell types, including neuroblasts, radial glia-like cells, astrocytes, OPCs, and ependymal cells. (B) Sketch illustrating the locations where we found neurogenic activity. (C) gt-SNE embedding of all cells from the relevant cell types shown in (A). The dashed line suggests the border between glia-like cells and neuroblasts. (D) Expression distribution of individual key genes projected onto the gt-SNE embedding. See also Figure S2.

**Figure S2:**
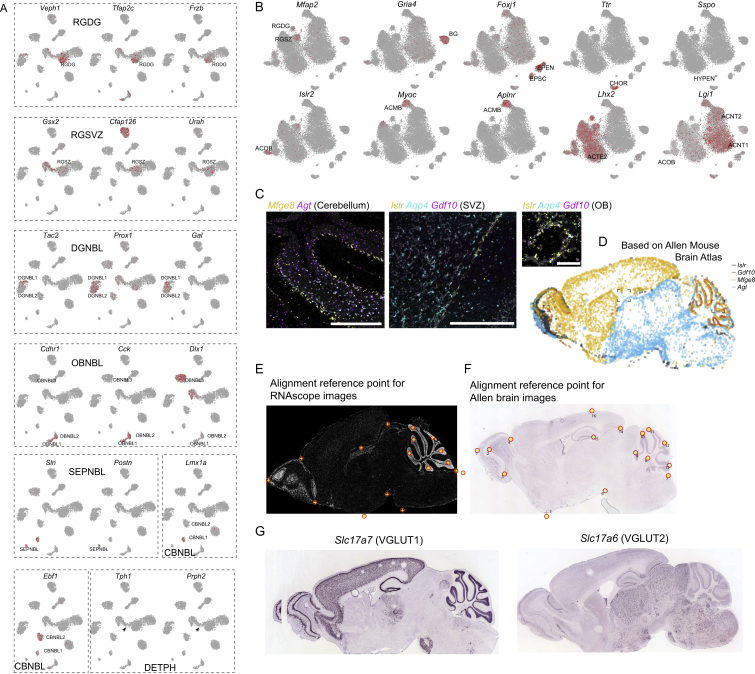
Markers and Validation of Neurogenesis and Astroependymal Cells, Related to Figure 2 (A) Additional marker genes for neurogenesis-related clusters, relevant clusters are indicated on the g-tSNE embedding. (B) Additional marker genes for various astroependymal cell types. The most enriched cluster is indicated. (C) Additional close-ups from validation using RNAscope. Genes and location indicated around the image. Scale bars: 500μm (CB, SVZ); 100μm (OB). (D) Composite image with colored dots representing cells, reconstructed from Allen Brain Atlas images, similar to Figure 3F but showing *in situ* hybridization. (E,F) Position of reference points used for alignment of multiple sagittal sections of RNAscope (E) and Allen Brain *in situ* hybridization (F) images. (G) *In situ* hybridization (Allen Brain Atlas) showing the extent of expression of *Slc17a6* and *Slc17a7*, for comparison with astrocyte cell types.

Although most neuronal types were already mature at the age investigated (postnatal days 20–30), we observed signs of ongoing neurogenesis in several regions (Figure 2). As expected, we detected the two regions that maintain adult neurogenesis in the mouse: the subventricular zone along the striatum and the dentate gyrus subgranular zone. In the subventricular zone, radial glia-like cells (RGSZ) and cycling neuronal intermediate progenitor cells (SZNBL) were linked to more mature and presumably migrating neuroblasts along the rostral migratory stream and in the olfactory bulb (OBNBL3). In the subgranular zone of the dentate gyrus, radial glia-like cells (RGDG), neuroblasts (NBDG), and immature granule cells (DGNBL1 and DGNBL2) would give rise to mature granule cells (DGGC), as recently described in detail (Hochgerner et al., 2018). The radial glia-like cells (RGSZ and RGDG), which are the stem cells of both lineages, expressed *Riiad1* (shared with ependymal cells; Figure 2D) and were more similar to astrocytes than to any neuroblast. Each local neurogenic niche was further marked by specific genes (Figure S2; e.g., the transcription factors *Tfap2c* in RGDG and *Urah* in RGSZ).

Neuroblasts across the brain fell into two general categories represented by two subtrees in the dendrogram (labeled “NBL” in Figure 1D). The first category expressed *Igfbpl1* and was either GABAergic (OBNBL3) or did not express any clear neurotransmitter phenotype. These neuroblasts were found in the rostral migratory stream (SZNBL and OBNBL3), dentate gyrus (DGNBL2), and habenula (DETPH). The second category expressed the T-box transcription factor *Eomes* (also known as *Tbr2*) and the vesicular glutamate transporter (*Slc17a7,* also known as VGLUT1) and thus was glutamatergic. These neuroblasts were found in the olfactory bulb (OBNBL1, OBNBL2), cerebellum (CBNBL), and septum (SEPNBL). However, *Eomes* and *Igfbpl1* overlapped in some populations (DGNBL1 and to some extent SZNBL), indicating that these categories of neuroblasts may represent sequential stages of neuronal maturation rather than divergent cell types. *Eomes*-expressing neuroblasts, with a generally less mature neurotransmitter phenotype, may then represent early stages of neuronal differentiation, whereas *Igfbpl1* spans both early and later stages, as was already shown in the dentate gyrus (Hochgerner et al., 2018).

### Astroependymal Cells Are Diverse and Spatially Patterned

**Figure 3:**
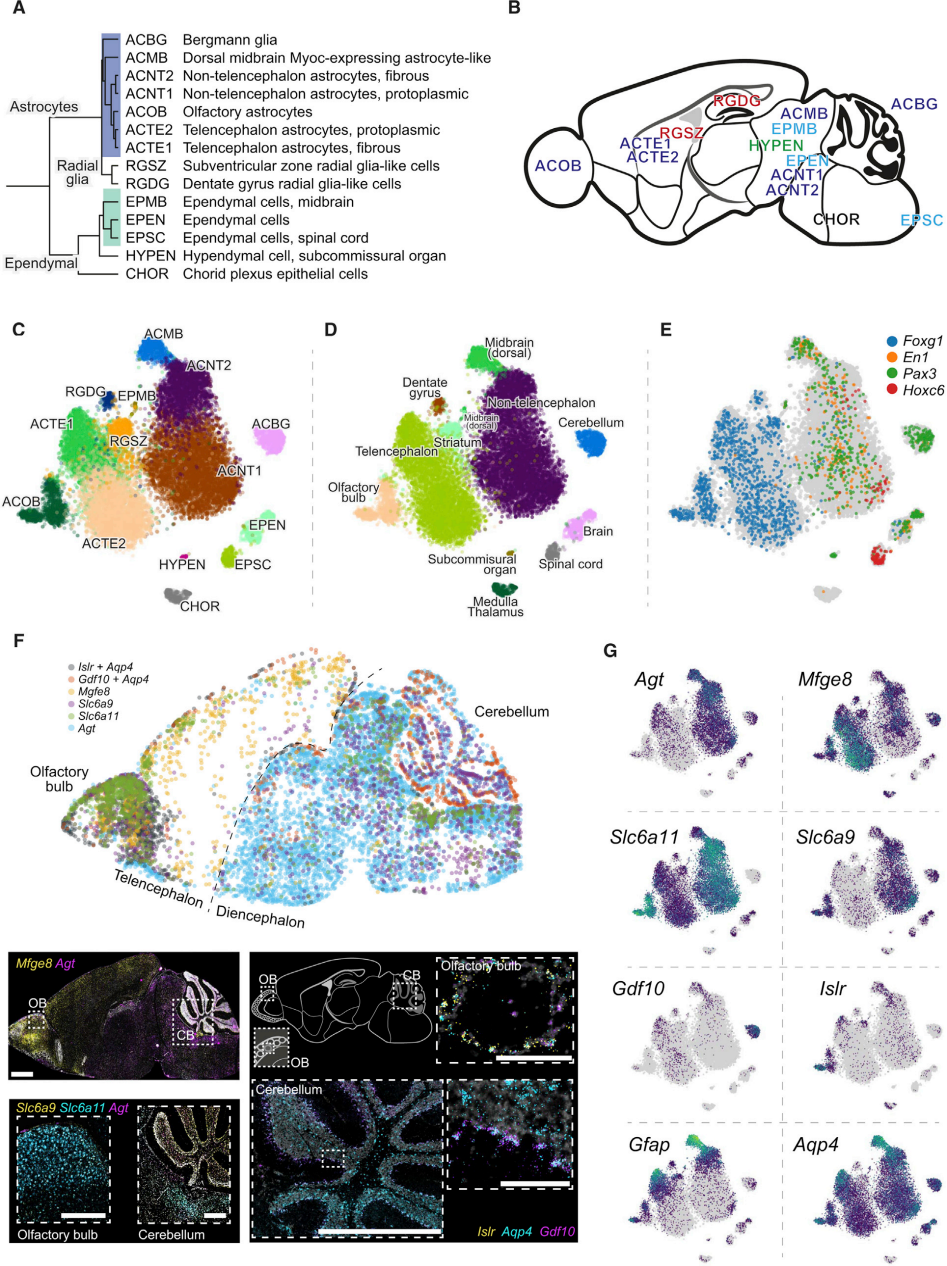
Molecular and Spatial Diversity of the Astroependymal Cells in the CNS (A) Subtree describing the hierarchy of astroependymal cell types. (B) Schematic sagittal section showing the location of astroependymal cells. (C–E) gt-SNE embedding of all cells from the relevant clusters colored by cluster identity (C), tissue of origin (D), and patterning transcription factors (E). (F) Validation of spatial distribution of astrocytes cell types using multiplex ISH (RNAscope). Images from three consecutive sections were aligned and overlaid (see STAR Methods) to generate a composite with dots representing cells (upper panel). Below, high-magnification images show details of spatial location. Scale bars: top and bottom left, 500 μm; right, 1000 μm (cerebellum overview) and 100μm (olfactory bulb and cerebellum zoom-ins). (G) Gene expression of selected markers shown on the gt-SNE layout.

Astrocytes, ependymal cells, and radial glia are developmentally related cell types and formed a subtree in the dendrogram (Figure 3). This taxon included two specialized secretory cell types: the hypendymal cells (HYPEN), which are specialized ependymal-like cells of the subcommissural organ that secrete SCO-spondin (encoded by *Sspo*) into the cerebrospinal fluid to form Reissner’s fiber, and the choroid plexus epithelial cells (CHOR), which are an extension of the ependymal lining of the ventricular surfaces that envelop branching capillaries protruding into the ventricles and secrete the extremely abundant thyroxine and retinol transport protein Transthyretin (encoded by *Ttr*).

Three types of ependymal cells all expressed *Foxj1*, the master regulator of motile cilia (Yu et al., 2008). The first, EPEN, was common along the rostrocaudal axis. The second, EPMB, was observed in the dorsal midbrain and—to a lesser extent—the hypothalamus. They expressed high levels of *Gfap* and the *Efnb3* gene encoding Ephrin B3 but only low levels of *Foxj1*. They also expressed many markers of tanycytes of the third ventricle, including *Nes*, *Vim*, *Rax*, and *Gpr50* (Miranda-Angulo et al., 2014), but their location in the dorsal midbrain suggests that they instead represented a tanycyte of the circumventricular organs (Kettenmann and Ransom, 2013). The third, EPSC, was specific to the spinal cord and was distinguished by the expression of immediate-early genes such as *Fos*, *Junb*, and *Egr1*.

Astrocytes were first described in 1858 by Rudolf Virchow, and in the second half of the 19^th^ century, a number of distinct astrocyte types were identified. With the exception of the radial astrocytes in neurogenic regions of the brain (see above), reactive astrocytes in response to injury, and types defined solely by morphology (such as velate astrocytes of the cerebellum and olfactory bulb), the modern understanding of astrocyte diversity essentially stands as it stood in 1900: the major types of mature astrocytes are believed to be the Müller glia of the retina, the Bergmann glia of the cerebellum, and the widely distributed protoplasmic and fibrous astrocytes (Ben Haim and Rowitch, 2017).

Here, we observed seven molecularly distinct types of astrocytes with clear regionally specialized distribution. All astrocytes expressed *Aqp4*, encoding aquaporin 4, the water channel located on astrocyte vascular endfeet. In addition to Bergmann glia of the cerebellum (ACBG), we found olfactory-specific astrocytes (ACOB, unrelated to olfactory ensheathing cells; see below), two subtypes of telencephalon-specific astrocytes (ACTE1 and ACTE2), two subtypes of non-telencephalon astrocytes (ACNT1 and ACNT2), and a *Myoc*-expressing astrocyte of the dorsal midbrain, ACMB. Müller glia were not observed because we did not sample from the retina.

We also did not observe distinct spinal cord populations corresponding to those previously described in the embryonic spinal cord (Hochstim et al., 2008). Those astrocytes were described as dorsoventrally patterned and distinguished by combinatorial expression of *Reln* and *Slit1*, but to our knowledge, they have not been more extensively characterized molecularly or at postnatal stages. We did not observe *Slit1* expression in astrocytes, while *Reln* expression correlated with *Gfap* (not shown).

Olfactory astrocytes were located around the olfactory glomeruli and could represent the previously described velate astrocytes, for which no molecular properties are known; they highly specifically expressed the *Islr* and *Islr2* genes encoding immunoglobulin-domain cell adhesion proteins (Figures 3 and S2), among other genes.

Telencephalon astrocytes ACTE1 and ACTE2 were distinguished by the expression of several genes, including *Mfge8* and *Lhx2*, and were found in the olfactory bulb, cerebral cortex, striatum, amygdala, and hippocampus but were absent from the hypothalamus, thalamus, midbrain, and hindbrain. Non-telencephalic astrocytes ACNT1 and ACNT2 showed the opposite distribution, marked by *Agt* (Angiotensinogen) and found in all regions caudal to the telencephalon/diencephalon border (i.e., posterior to and including the hypothalamus and thalamus). The border between the two was sharp, as judged by ISH of the relevant genes (Figure S2D), indicating that they do not intermingle across substantial distances.

We validated the identity and distribution of astrocyte cell types using RNA FISH (RNAscope), which was consistent with the Allen Mouse Brain Atlas (Figures 3F and S2). Co-staining of *Mfge8* and *Agt* on a sagittal section revealed a distinct border separating the telencephalon from the diencephalon. The olfactory bulb and cerebellum were enriched with their local astrocytes ACOB and ACBG marked by *Islr* and *Gdf10*, respectively. Moreover, we validated the distribution of neurotransmitter transporters with *Slc6a11* (also known as GAT3, the GABA reuptake transporter), highly expressed in the olfactory and the non-telencephalon astrocytes (but not in cerebellum), and *Slc6a9* (glycine transporter GLYT1), with similar pattern but lower olfactory expression and a higher expression in the cerebellum.

Both telencephalon and non-telencephalon astrocytes were further split into subtypes expressing *Gfap* at high or low levels (Figure 3G). This distinction likely corresponds to the fibrous astrocytes of the white matter and the *glia limitans* underneath the pia (*Gfap*-high) versus the protoplasmic astrocytes of the parenchyma (*Gfap*-low). The difference between subtypes in both cases involved a similar set of genes, suggesting that this represents an independent axis of variation that can be activated in both telencephalon and non-telencephalon astrocytes as a function of local environmental cues, particularly the distance from the pia and white matter.

Interestingly, like neurons, these diverse astrocyte and ependymal cell types occupied distinct domains of the brain with little apparent mixing. The sharpness of the border between *Mfge8* (telencephalon astrocytes) and *Agt* (non-telencephalon atroctyes) expression, for example, and the fact that it coincided with a developmentally recognized boundary distinguishing the telencephalon from the rest of the brain, strongly implies that these astrocyte types are developmentally specified. In order to test this hypothesis, we examined the expression of region-specific neural tube patterning genes, the transcription factors *Foxg1* (telencephalon), *En1*, and *Pax3* (midbrain) and *Hoxc6* (spinal cord). Each of these genes marked the expected subset of astrocyte and ependymal cell types (Figure 3G). Thus, we have uncovered a diversity of astrocyte and ependymal cell types, showing the hallmarks of developmentally specified identities and regional specialization.

We can only speculate as to the functional distinction between telencephalic and non-telencephalic astrocytes. Given the important role of astrocytes in maintaining neurotransmission, it’s striking that the distinction between telencephalic and non-telencephalic astrocytes coincided with the prevalence of VGLUT1 in the telencephalon versus VGLUT2 in the di-/mesencephalon and hindbrain (Figures S1C and S2G; however, the thalamus used both VGLUTs). This indicates a possible role in maintaining distinct modes of glutamatergic neurotransmission.

Furthermore, one of the genes most highly enriched in non-telencephalic astrocytes was *Slc6a9* (Figure 3G), encoding the glycine reuptake transporter GLYT1. Glycine is a widely used inhibitory neurotransmitter only in the caudal parts of the brain and in the spinal cord. This suggests a specific role for non-telencephalic astrocytes in clearing glycine from the synaptic cleft. We note that since GLYT1 is a reversible glycine transporter (Supplisson and Roux, 2002), if it were expressed in telencephalic astrocytes, then those cells would potentially secrete glycine into the synaptic cleft instead of absorbing it. Glycine is not only an inhibitory neurotransmitter, but also a co-ligand for the NMDA glutamate receptor involved in coincidence detection. This may explain the need for distinct types of astrocytes in glycine-rich and glycine-poor regions of the brain.

### Loss of Patterning in the Oligodendrocyte Lineage and Convergence to a Single Brain-wide Intermediate State

**Figure 4:**
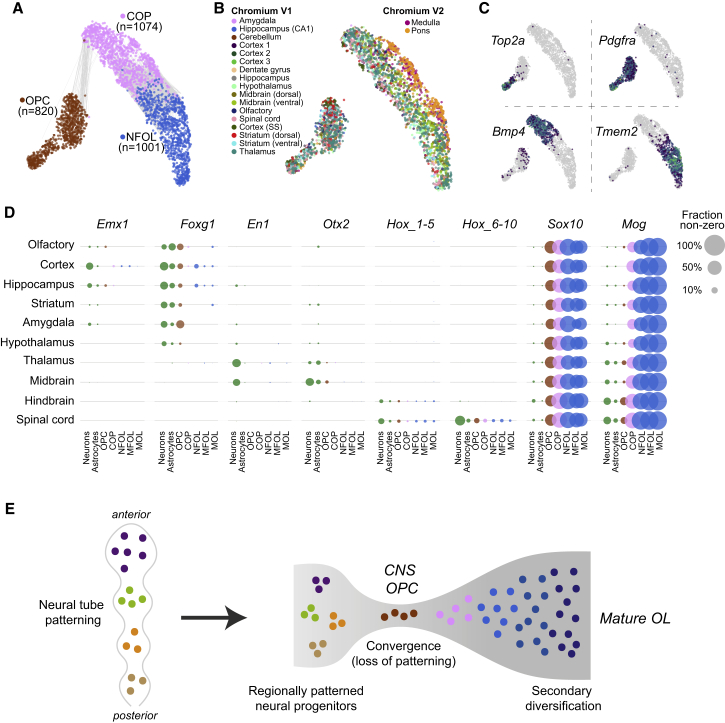
Convergence to a Common State at the Early Stages of Oligodendrocytes Lineage (A and B) gt-SNE embedding of the three first stages of the oligodendrocytes lineage OPC, COP, and NFOL colored by cluster identity (A) and tissue of origin (B). Edges in (A) connect nodes between mutual neighbors (*k* = 150), but only if they are from different clusters. (C) Gene expression of selected markers overlaid on gt-SNE embedding. (D) Patterning transcription factor analysis. Circles represent fraction of positive cells in each cluster and brain region. (E) Illustration of the proposed model of primary patterning, loss of regional identity and secondary diversification.

Oligodendrocytes wrap myelin sheets around axons to support long-range neurotransmission and were previously analyzed by scRNA-seq (Marques et al., 2016). The greatly increased sampling depth in the present study did not reveal any additional, clearly distinct subtypes of mature oligodendrocytes beyond those we had already described. Furthermore, OPCs remained a single cluster, with only a distinction between cycling and non-cycling OPCs (Figures 4A and 4B). Immature oligodendrocyte lineage types (OPC, committed oligodendrocyte precursor [COP], and newly formed oligodendrocyte [NFOL]) were intermingled in all tissues (except for a technical batch effect in the medulla and pons; Figure 4B). Note that although the oligodendrocyte lineage clusters aligned well with those previously published (Figure S1H; Marques et al., 2016), there was not a perfect correspondence, and subtype labels in this paper do not correspond exactly to those previously described.

Thus, regardless of the tissue sampled, OPC, COP, and NFOL presented as single, brain-wide common cell types. Since OPCs are the progenitors of the entire oligodendrocyte lineage, this observation demonstrates that the diversity observed among mature oligodendrocytes (Table S3) must be the result of a secondary diversification, not developmental patterning. Oligodendrocyte morphology varies according to the type of axon they myelinate, but transplantation experiments indicate that those differences are plastic (Richardson et al., 2006). This may also explain the graded, interspersed pattern of diversity among mature oligodendrocytes, in contrast to the division into cell types with clear boundaries (molecularly and anatomically) that we observed among astrocytes and neurons.

OPCs are derived from neural tube precursors that are patterned along the anteroposterior axis. This has been demonstrated clearly, e.g., by genetic lineage tracing of *Emx1*-positive neural progenitors, which selectively labels forebrain oligodendrocytes (Kessaris et al., 2006). Thus, at some point, cells that later become OPCs must have been molecularly distinct along the anteroposterior axis, for example, expressing *Emx1* in the forebrain, *En1* in the midbrain, and *Hox* genes in the hindbrain and spinal cord. Yet this did not translate into distinct OPC types along the same axis. Clearly, at some point, anteroposterior patterning must be lost in the oligodendrocyte lineage. We therefore asked if, despite the lack of clearly distinct subtypes, OPCs, COPs, or NFOLs sampled from different tissues retained any traces of patterning gene expression (Figure 4D). We confirmed induction of the key transcription factor *Sox10* in OPCs and of the myelin oligodendrocyte glycoprotein *Mog* in COPs. In the spinal cord, we detected a clear expression of *Hox* genes 6–10 (that is, *Hoxa6*, *Hoxb6*, *…*, *Hoxd10*), which are responsible for patterning the thoracic spinal cord. These genes were expressed in spinal cord oligodendrocytes only, at levels similar to those observed in neurons and astrocytes, and they remained expressed throughout the oligodendrocyte lineage. Similarly, we found that *Hox* genes 1–5 (that is, *Hoxa1*, *Hoxb1*, *…*, *Hoxc5*) were expressed in the hindbrain and spinal cord oligodendrocytes, remained expressed into mature oligodendrocytes, and were expressed at levels similar to those in neurons and astrocytes.

In contrast, several transcription factors responsible for patterning the forebrain and midbrain were detected only at very low levels (<1% of cells) if at all and did not appear in mature oligodendrocytes. At such low levels of expression, it is difficult to rule out a contamination from adjacent neurons, and thus, it is possible that they were not expressed at all in the oligodendrocyte lineage.

We conclude that OPCs (and, to a lesser extent, COPs and NFOLs) may retain a memory of their anteroposterior position in the form of expression of region-specific transcription factors but that this does not translate into clearly distinct region-specific cell types. Thus, an initially diverse population of neural progenitors converges on a single intermediate transcriptional state (OPC or COP) and is then subject to secondary diversification as they mature (Figure 4E). Similar phenomena were recently reported in embryonic stem cells *in vitro* (Briggs et al., 2017) and, less dramatically, in the developing *Drosophila* brain (Li et al., 2017).

### Vascular Cells and a Family of Broadly Distributed Mesothelial Fibroblasts

**Figure 5:**
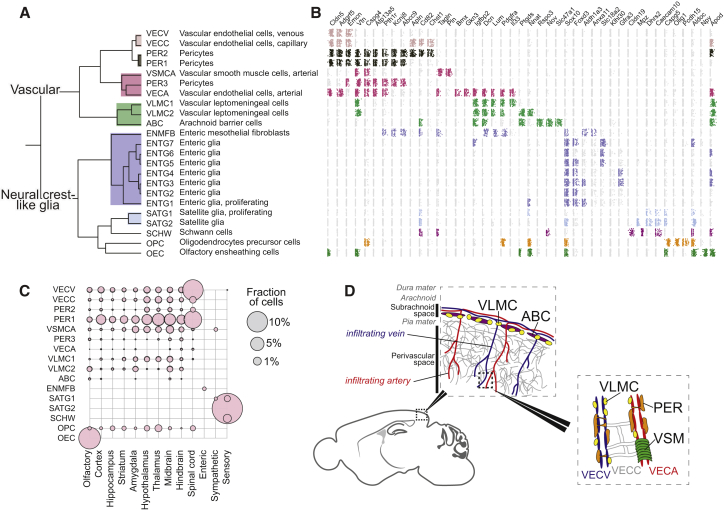
Diversity of the Vasculature and Neural-Crest-like Glia (A) Subtree describing the vasculature and neural-crest-like glia. (B) Expression dot plots for marker genes on log scale and jittered vertically in a uniform interval. Dots are colored only if the trinarization score is positive (posterior probability greater than 0.95), and colors represent the taxonomy rank 4 taxa. (C) The tissue contribution to each cluster represented by the circle size (enteric glia not shown). (D) Schematic illustration of the approximate position of vascular cell types and the meninges. See also Figure S3.

A recent paper characterized vascular cells across the murine brain (Vanlandewijck et al., 2018), describing twelve vascular cell types. Like Vanlandewijck et al., we observed (Figure 5) distinct endothelial cell types carrying known arterial (e.g., *Bmx*; VECA) and venous (*Slc38a5*; VECV) markers, as well as capillary endothelial cells (VECC) expressing *Meox1*. We found three types of pericytes and a single arterial vascular smooth muscle type (*Acta2*, *Tagln*; VSMCA). Based on the proportion of all cells that were vascular, the mid- and hindbrain and spinal cord were the most vascularized (Figure 5C).

**Figure S3:**
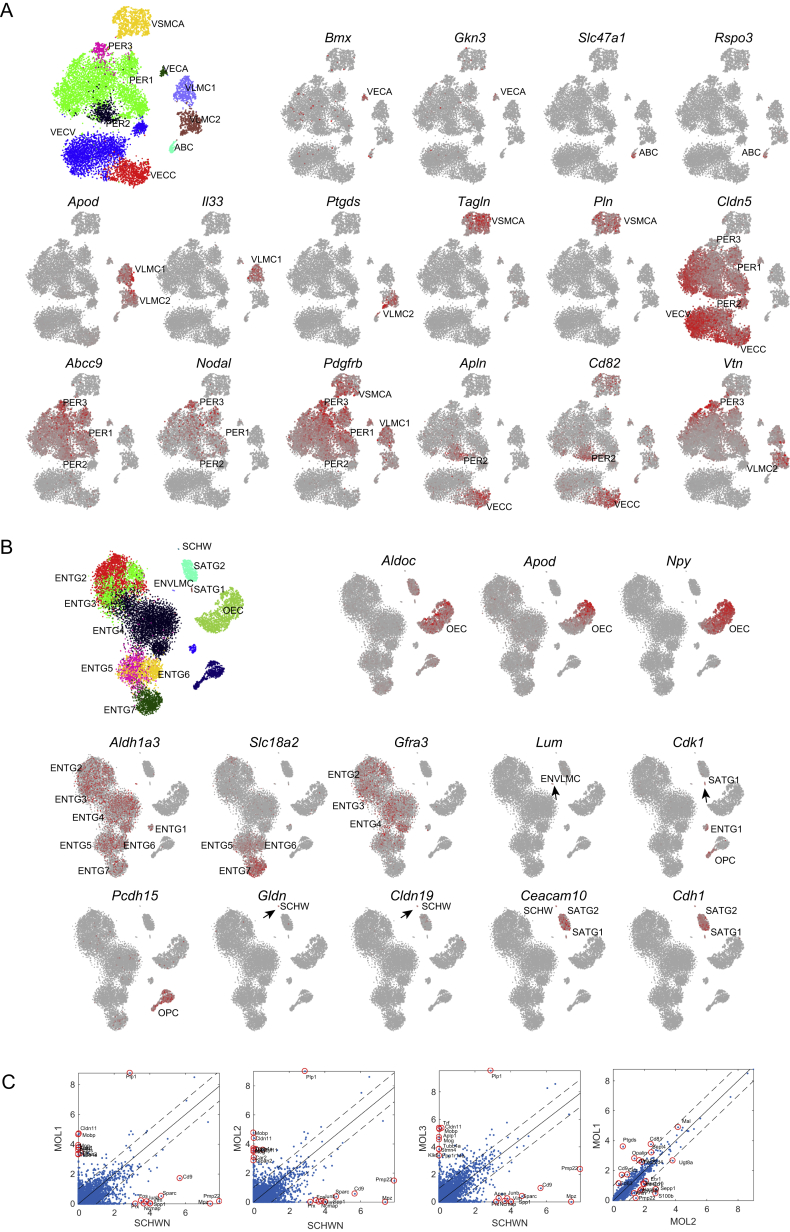
Markers of Neural-Crest-like Glia and Vascular Cell Types, Related to Figure 5 (A) Additional marker genes for the neural crest-like glia taxonomy unit, related to Figure 5. First panel on the left show the different clusters. Other panels show the expression (red high) distribution of marker genes. Black arrows indicate small clusters. (B) Similar to (A) but for the vasculature taxon. (C) Scatterplot showing differences between Schwann cells and mature oligodendrocytes (MOL) clusters. Values shown are log_2_(x+1) transformed average molecule counts. The top 10 differentially expressed genes are shown in red and labeled.

Vanlandewijck et al. described two brain fibroblast-like cell types expressing fibril-forming collagens (e.g., *Col1a1*, *Col1a2*), collagen fiber crosslinking proteins (*Lum*, *Dcn*), and the platelet-derived growth factor receptor alpha, *Pdgfra*, which was interposed between astrocyte endfeet and vascular endothelial cells. Brain fibroblast-like cells are likely identical to the vascular leptomeningeal cells (VLMCs) that we previously described in the mouse CNS (Marques et al., 2016). In the present dataset, we observed four types sharing the canonical markers. Two types were distinguished by expression of genes including the pro-inflammatory cytokine *Il33* (VLMC1) and the Prostaglandin D2 synthetase *Ptgds* (VLMC2), the latter previously found as the most highly enriched gene in mouse leptomeninges (Yasuda et al., 2013) (Figure S3A).

Furthermore, we discovered two additional related cell types that shared expression of the canonical VLMC markers. We identified one (ABC) as arachnoid barrier cells based on the expression of *Abcg2* and *Pgp*. These two genes encode drug and xenobiotic transporters known as BCGP and P-gp, respectively, which are expressed on barrier cells of the arachnoid mater of the meninges (Yasuda et al., 2013). The most specific gene expressed in ABCs was *Slc47a1*, which encodes the multidrug and toxin extrusion protein MATE1, reinforcing the putative function of ABCs to cleanse the cerebrospinal fluid of toxic substances. In contrast to all other VLMC-like cell types, ABCs did not express *Lum* and showed only very low levels of *Pdgfra*.

The fourth VLMC-like cell type (enteric mesothelial fibroblasts; ENMFBs) expressed all the VLMC marker genes but was found exclusively in the enteric nervous system. This demonstrates that VLMC-like cells are present throughout the body and are not brain specific. Like the brain, organs of the abdomen are wrapped in protective layers of cells, which serve protective, lubricating, and active signaling functions, especially during development. Our observations thus support the view that VLMC-like cells are a family of functionally related (but organ-specific) mesothelial fibroblasts that form protective membranes around internal organs, including the pia and arachnoid membranes of the brain. The fact that ENMFBs were obtained by sorting *Wnt1-Cre;R26Tomato* cells indicates that these cells are derived from the neural crest, as has previously been shown for both pia and arachnoid.

### Neural-Crest-Derived Glia and Oligodendrocyte Progenitors

A subtree of the dendrogram in Figure 1 comprised peripheral glia—seven types of enteric glia (ENTG1-7), proliferating (SATG1), and non-proliferating (SATG2) satellite cells of the sensory and sympathetic nervous system and Schwann cells (SCHW)—along with olfactory ensheathing cells (OECs) and OPCs of the CNS.

Satellite glia cover the surfaces of sensory and sympathetic neurons and are thought to support their function, but in unknown ways. Satellite glia were enriched in transporters of amino acids (*Slc7a2*), purine nucleobases (*Slc43a3*), and long-chain fatty acids (*Slc27a1*), indicating a role in supporting the metabolism of neurons.

The diversity and function of enteric glia are not known in detail. Enteric glia were very abundant in our dataset (91% of all enteric cells) and almost as diverse as enteric neurons, with seven distinct types. One type (ENTG1) was proliferating (expressing *Top2a*) and could represent a progenitor type. Intriguingly, some enteric glia expressed the vesicular monoamine transporter *Slc18a2* (Figure S3B), which otherwise loads monoamine neurotransmitters into synaptic vesicles in neurons.

Olfactory ensheathing cells are neural-crest-derived (Barraud et al., 2010) cells that ensheath axons of the olfactory sensory neurons but do not form myelin. Molecularly, they showed a peculiar combination of markers otherwise archetypical of oligodendrocytes (*Plp1*, *Sox10*), pericytes (*Vtn*), endothelial cells (*Cldn5*), neurons (*Npy*), and astrocytes (*Aldoc*), as shown in Figure S3B.

Schwann cells are the myelinating glia of the PNS and differ from CNS oligodendrocytes by expressing myelin components *Pmp22* and *Mpz*, the tetraspanin *Cd9*, and the extracellular matrix calcium-binding osteonectin *Sparc* and by not expressing the tight junction *Cldn11*, the myelin protein *Mobp*, *Mog*, and the iron chelator *Trf* genes (Figure S3C).

In contrast to all other cell types of this taxon, which are neural-crest derived, OPCs are derived from the neural tube and assumed to be produced by the same progenitors as astrocytes and neurons. Interestingly, however, OPCs share many features of neural crest cells: they require the expression of the two key transcription factors that specify neural crest (*Sox10* and *Sox9*) (Takada et al., 2010), they are highly migratory, and they do not respect developmental borders in the brain. These observations, and the finding that OPCs align molecularly with all the neural-crest-derived glia, suggest that they are a neural-crest-like type of glia and support the view that they have a common evolutionary origin with Schwann cells (Kastriti and Adameyko, 2017). Although they are not derived from the physical neural crest, they appear to use similar regulatory mechanisms as neural-crest-derived cells. We therefore named this taxon “neural-crest-like glia.”

### Peripheral Nervous System

**Figure S4:**
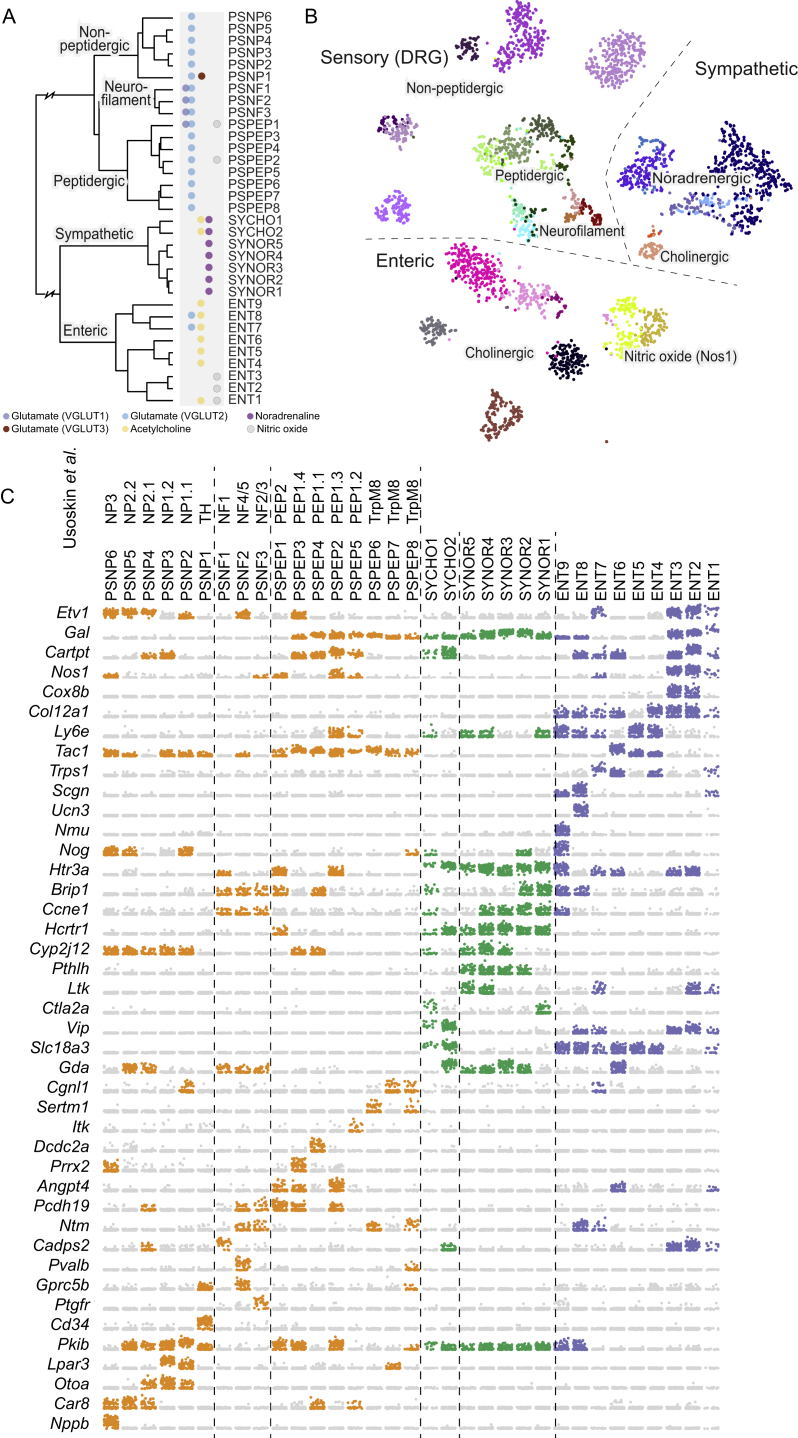
Neurons of the Peripheral Nervous System, Related to Figure 7 (A) Hierarchical structure of the peripheral nervous system neuronal cell types. Neurotransmitters used by each cell types are indicated by the colored dots next to each leaf. (B) gt-SNE embedding of all related cells demonstrate the diversity and abundance of the different clusters. (C) Dot plots for marker genes along the PNS neurons. Dots show gene expression on log scale, and jittered vertically for clarity. Colors are shown only if the trinarization score is positive (posterior probability greater than 0.95 with *f = 0.2*).

Neurons of the PNS segregated molecularly from the CNS and formed distinct sensory, sympathetic, and enteric subdivisions (Figures 1C and S4). Within the peripheral sensory neurons (of the dorsal root ganglia), cell types were divided into three main branches: peptidergic (eight types), non-peptidergic (six types), and neurofilament (three types), which suggests refinements to previous classifications (Li et al., 2016, Usoskin et al., 2015) (Figure S4 and Table S2).

Within the sympathetic ganglia, we found two cholinergic and five noradrenergic cell types in agreement with our previous classification (Furlan et al., 2016) (Figure S4 and Table S2).

The enteric nervous system has not been previously studied in molecular detail using single-cell methods. Here, we report on the composition of the myenteric plexus of the small intestine, whereas we did not include cells from the submucosal layer or other regions of the gastrointestinal tract. Based on marker gene expression, morphology, location, and projection targets, approximately ten cell types have been previously described (Furness et al., 2014, Qu et al., 2008) in the myenteric plexus of the mouse.

We found nine molecularly distinct neuron types. Although enteric neurons are commonly divided into nitrergic and Calretinin-expressing subtypes, our data indicate that the more natural split is between nitrergic (i.e., expressing the neuronal nitric oxide synthase *Nos1*, ENT1-3) and cholinergic (i.e., expressing *Chat* and *Slc5a7*, ENT4-9) neurons. The nitrergic ENT1-3 expressed the high-affinity choline transporter *Slc5a7*, but *Chat* was very low or undetected in those neurons. In addition, ENT7 and ENT8 co-expressed *Slc17a6* (VGLUT2). Many enteric neurons also expressed a variety of neuropeptides (Figure S4), including *Gal*, *Cartpt*, *Nmu*, *Vip*, *Cck*, and *Tac1*. A more detailed analysis of these neurons will be published elsewhere (U.M., unpublished data).

### Central Nervous Systems Neurons

Telencephalon-projecting neurons (expressing high *Ptk2b*, *Ddn*, *Icam5*) formed a distinct set of 32 clusters including pyramidal cells of the cortex and hippocampus, as well as medium spiny neurons (MSNs) of the striatum. The cerebral cortex was the most diverse, with 20 projection cell types, which were glutamatergic (all VGLUT1, but some additionally VGLUT2 or VGLUT3). Closely related were the hippocampal pyramidal cells (three types) and the dentate gyrus granule cells, as well as a single cluster from the basolateral amygdala, all of which—like the isocortex—develop from the pallium.

The GABAergic MSNs of the striatum are classified as D1 type or D2 type according to the dopamine receptor they express. A longstanding question concerns the diversity of these cell types, particularly relative to structural and functional features of the striatum. For example, dorsal MSNs initiate and control movements, whereas ventral MSNs are involved in motivation, reward, aversion, and similar behaviors. We found two D1-type MSNs (MSN1 and MSN4), one enriched in dorsal and one in ventral striatum, as well as two D2-type MSNs (MSN2 and MSN3), also dorsal and ventral, demonstrating a molecular distinction corresponding to the distinct circuits and functions of dorsal and ventral MSNs. In addition, we found putative patch-specific D1-/D2-type neurons (expressing *Tshz1*) and matrix-specific D2 neurons (expressing *Gng2*), consistent with staining patterns of these genes in the Allen Mouse Brain Atlas.

Telencephalic inhibitory interneurons, including cells from the olfactory bulb, cortex, hippocampus, and thalamus, formed a separate taxon, with the olfactory cells as a distinct subgroup. The thalamic inhibitory neurons expressed *Meis2* and shared key transcription factors (e.g., *Dlx1*, *Dlx2*, *Dlx5*, *Dlx6*) with other cell types in this taxon, as well as with striatum MSNs, consistent with a common developmental origin in the ganglionic eminences.

Most olfactory neurons were GABAergic, in agreement with previous work (Nagayama et al., 2014), and one was also dopaminergic. We found no mature glutamatergic neurons in the olfactory bulb. However, two neuroblast types (OBNBL1 and OBNBL2), putatively located in the mitral cell layer, may represent immature versions of the olfactory projection neurons, the mitral, and tufted cells. One of them, OBNBL1, expressed the identifying marker of mitral cells, the T-box transcription factor *Tbx21*.

A single taxon collected nearly all cholinergic, monoaminergic neurons (which we identified based on expression of the necessary biosynthesis enzymes and vesicular and reuptake transporters; Figure S6C and STAR Methods) from the whole brain, as well as peptidergic neurons mainly of the hypothalamus. These included the cholinergic afferent nuclei of cranial nerves III-V (HBCHO4) and VI-XII (HBCHO3), the adrenergic nucleus of the solitary tract (HBADR), the noradrenergic cell groups of the medulla (HBNOR), five serotonergic hindbrain types, two dopaminergic neuron types of the ventral midbrain, and 15 types of peptidergic neurons, including those secreting neurotensin (*Nts*), vasopressin (*Avp*), oxytocin (*Oxt*), gonadotropin-releasing hormone (*Gnrh*), galanin (*Gal*), enkephalin (*Penk*), orexin (*Hcrt*), CART peptides (*Cartpt*), thyrotropin (*Trh*), pro-opiomelanocortin (*Pomc*), agouti-related peptide (*Agrp*), and neuromedin (*Nmu*) (Lam et al., 2017, Romanov et al., 2017). Most of these peptidergic cell types were located in hypothalamus, but some were from telencephalon (bed nuclei of stria terminalis and septal nucleus), midbrain (Darkschewitz nucleus), and spinal cord (central canal neurons; see below). The fact that the majority of cholinergic, monoaminergic, and peptidergic neurons clustered together suggests a common underlying regulatory state distinct from that in neurons using canonical neurotransmitters. On the other hand, they still retained their CNS neuron character and did not intermingle with cholinergic or monoaminergic neurons of the PNS.

We further found 38 excitatory and inhibitory cell types of the diencephalon (thalamus and hypothalamus) and midbrain, forming a unified taxon. These types segregated nearly perfectly into glutamatergic (mostly VGLUT1) and GABAergic subsets but included two cholinergic types (of the red nucleus and the habenula). The thalamus proper contained only glutamatergic neurons, except for the *Meis2*-expressing neurons of the reticular nucleus that forms a capsule around the thalamus. In the midbrain, the superior and inferior colliculi were the most diverse, comprising 17 excitatory (exclusively VGLUT1) and inhibitory (GABA) cell types, showing distinct spatial distributions. In the ventral midbrain, we identified two types of dopaminergic neurons, one cholinergic and four GABAergic.

In the hindbrain (15 types not including cerebellum), all inhibitory neurons were glycinergic (GLYT1, GLYT2, or both), and excitatory neurons were a mix of VGLUT1 and VGLUT2. We identified six cell types in the cerebellum, of which five are previously known: Purkinje cells, granular cells, granular layer interneurons, molecular layer interneurons, and granular cell neuroblasts. A sixth cell type (MEINH1), curiously, was found in the midbrain but was molecularly indistinguishable (Figure S1E) from cerebellum molecular layer interneurons (CBINH1). It is the only example of a neuronal cell type found in two different and distant regions.

Finally, in the spinal cord, we identified 22 cell types, again split into inhibitory (GABAergic or glycinergic) and glutamatergic (VGLUT2) in good agreement with an independent experiment focused on the dorsal horn (Häring et al., 2018) (Table S2). In addition, here we identified central canal neurons (SCINH11), known as cerebrospinal fluid-contacting neurons, which expressed transcription factors *Gata2* and *Gata3* (Figure S7) (Petracca et al., 2016). They also specifically expressed polycystin-like genes (*Pkd1l2* and *Pkd2l1*), which encode a mechanosensory protein complex that detects fluid flow, and *Espn*, which encodes an actin bundling protein with a major role mediating sensory transduction in mechanosensory cells. Thus, central canal neurons are likely specialized cells that monitor cerebrospinal fluid flow.

### Spatial Distributions Reflect Molecular Diversity

Given the importance of location for neuronal function, we wanted to assign a spatial distribution to each cell type. The Allen Mouse Brain Atlas provides systematic high-quality information about gene expression based on ISH. The data are available both as images and in the form of three-dimensional volumetric maps. We computed the spatial extent of each cell type by correlating volumetric and RNA-seq gene expression using only cell-type-specific genes as determined by a significant enrichment score (STAR Methods). The resulting data were visualized as three-dimensional density maps, expected to peak in regions where each cell type was abundant.

**Figure S5:**
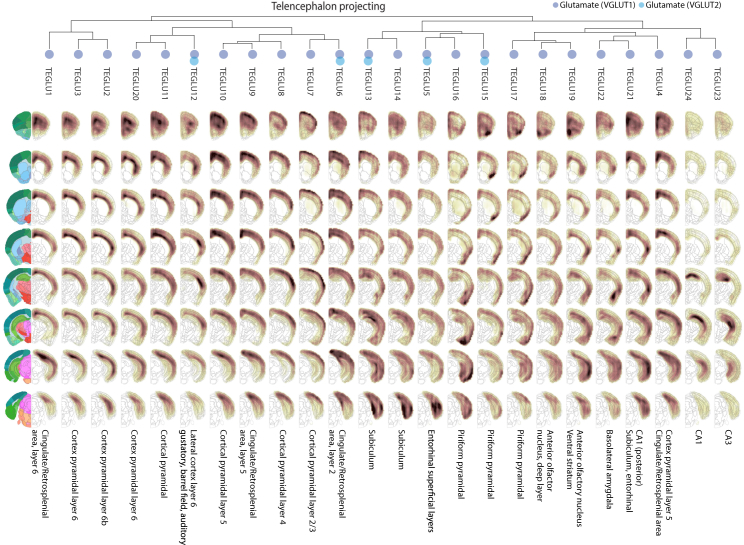
Spatial Distribution of All Telencephalon Excitatory Projecting Neurons, Related to Figure 6 Dendrogram above shows the hierarchical structure as in Figure 1C. Left, reference atlas annotation (Allen Brain Atlas). Each column shows the expression map of an individual cluster, where dark brown is high and white is low correlation.

Inspecting the resulting cell-type distribution maps, we found reassuringly that the automatically assigned locations corresponded well with the known source of the cells. For example, cortical and hippocampal projection neurons were assigned to the cortex and hippocampus as expected (Figure S5). But the spatial maps provided much more detail: for example, the distinction between CA1 and CA3 pyramidal cells was clear (Figure S5, right), and cortical pyramidal cells could be assigned highly specific distributions across the cortical surface (Figure S5, left) and layers. Interestingly, the spatial distribution of cortical pyramidal neurons correlated with their molecular similarity. For example, pyramidal neurons of the piriform and entorhinal cortex, as well as the subiculum, were molecularly closely related (shown by their forming a separate subtree of the dendrogram), as well as spatially aligned. Similarly, the pyramidal cells of the neocortex were arranged by molecular similarity in layer order (i.e., layers 2/3, layer 4, layer 5, layers 6/6b). Notably, this also corresponds with their order of development during embryogenesis.

**Figure 6:**
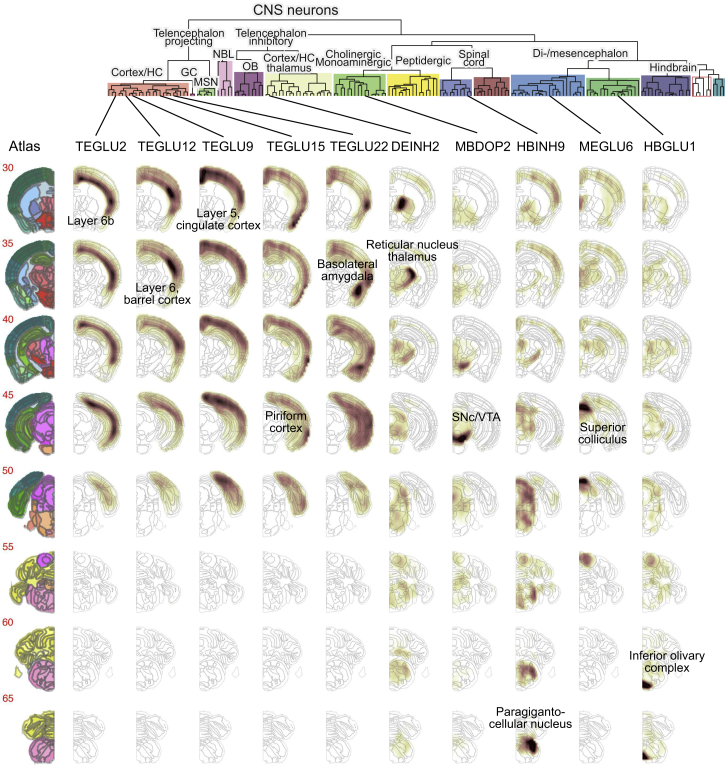
Neuronal Cell Types Are Spatially Restricted Examples of inferred spatial distributions for cell types across the brain. The left column shows reference images from the Allen Brain Atlas. Each row shows one coronal section, ordered rostrocaudally, and each column shows one cluster as indicated at the top. For every cluster and every voxel, the correlation coefficient is depicted by the colormap (dark high, white low). Labels indicate the top-scoring anatomical unit for each cluster. See also Figure S5.

Beyond the cortex, many cell types were assigned to very specific locations, greatly aiding interpretation of the data. For example, midbrain dopaminergic neurons (MBDOP2) were found in the substantia nigra and ventral tegmental area (Figure 6). Spatial distribution maps are provided for all CNS neurons at the companion wiki web site.

### Drivers of Neuronal and Glial Diversity

**Figure S6:**
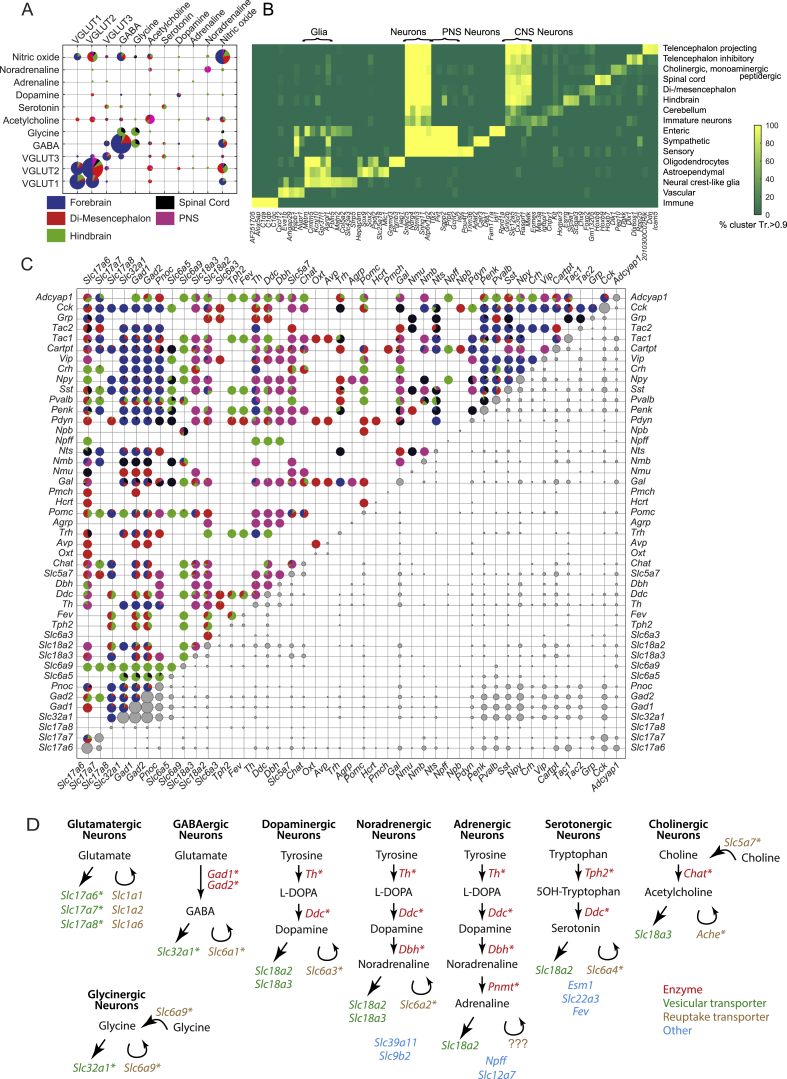
Neurotransmitter Modularity, Related to Figure 7 (A) Co-expression of neurotransmitters. Rows and columns represent neurotransmitters. Circle size represents the number of cells in clusters with the indicated combination. Each circle shows (as a pie chart) the brain compartment in which we found the relevant clusters. (B) Analysis of pan-markers along the different ranks of the taxonomy. The heatmap represents the percentage of clusters with trinarization score greater than 90% within the taxonomy unit. Rows represent taxonomy units and columns genes. (C) Similar co-expression analysis as in (A) but with individual genes encoding neurotransmitters and neuropeptides. Upper half of the matrix shows pie charts representing only the compartment distribution. Lower part of the matrix represent the frequency each combination was found. (D) Summary diagram of the biosynthesis components and transporters used to define neurotransmitter phenotypes. Asterisks indicate the genes used separately (glutamatergic neurons) or jointly (all other) to identify each class of neurotransmitter.

In order to better understand the forces that drive gene expression diversity in the mammalian nervous system, we next examined the expression of neurotransmitters and neuropeptides. We examined the co-expression of neurotransmitters while retaining information about the tissue compartment (Figure S6A). While glutamate (VGLUT2) was the only neurotransmitter expressed in all compartments, GABA contributed the larger number of cell types and was mostly concentrated in the forebrain. GABAergic and glutamatergic (VGLUT1 and VGLUT2) neurotransmission was mutually exclusive; we did not find a single cell type anywhere in the nervous system that expressed both. Glutamatergic neurons in the telencephalon all used VGLUT1, with some additionally using VGLUT2, whereas in more caudal regions, VGLUT2 dominated. Interestingly, the boundary that separated VGLUT1 dominance from VGLUT2 dominance appeared to be the telencephalon-diencephalon border, analogous to the separation of the two major types of astrocytes at this same boundary (although both were expressed in the thalamus).

In contrast, the atypical vesicular glutamate transporter VGLUT3 was often co-expressed (Figures 1C and S6A) with cholinergic and monoaminergic neurotransmitters and more rarely alone or with the other VGLUTs or GABA. This supports the notion that VGLUT3 plays a distinct role in cell types that release non-canonical neurotransmitters. Acetylcholine occurred with most other neurotransmitters, whereas, for example, serotonin occurred only alone or with VGLUT3 or GABA. The gaseous neurotransmitter nitric oxide (i.e., *Nos1* expression) was detected throughout the nervous system and did not combine preferentially with (or avoid) any other neurotransmitter (Figures 1C and S6A).

Examining the co-expression matrix of individual genes encoding neurotransmitter enzymes, vesicular and reuptake transporters, and neuropeptides, we found stereotyped combinatorial patterns assigned to specific compartments of the nervous system (Figure S6C). This analysis demonstrates how the rules governing gene co-expression can vary between brain regions. For example, somatostatin (*Sst*) is a canonical marker of inhibitory neuronal subtypes in the forebrain but was widely expressed in excitatory neurons in the spinal cord, hindbrain, and di-mesencephalon. Moreover, *Sst* was also expressed in combination with *Fev* (serotonin, hindbrain), *Dbh* (noradrenalin, PNS), or the neuropeptide *Trh* (hypothalamus). In contrast, *Pvalb*—another canonical marker of forebrain inhibitory cells—was also expressed in excitatory neurons in the mid- and hindbrain. These results demonstrate that neuropeptides, neurotransmitters, calcium-binding proteins, and other neuronal molecules are used in a highly modular fashion and serve different functions in different contexts.

**Figure 7:**
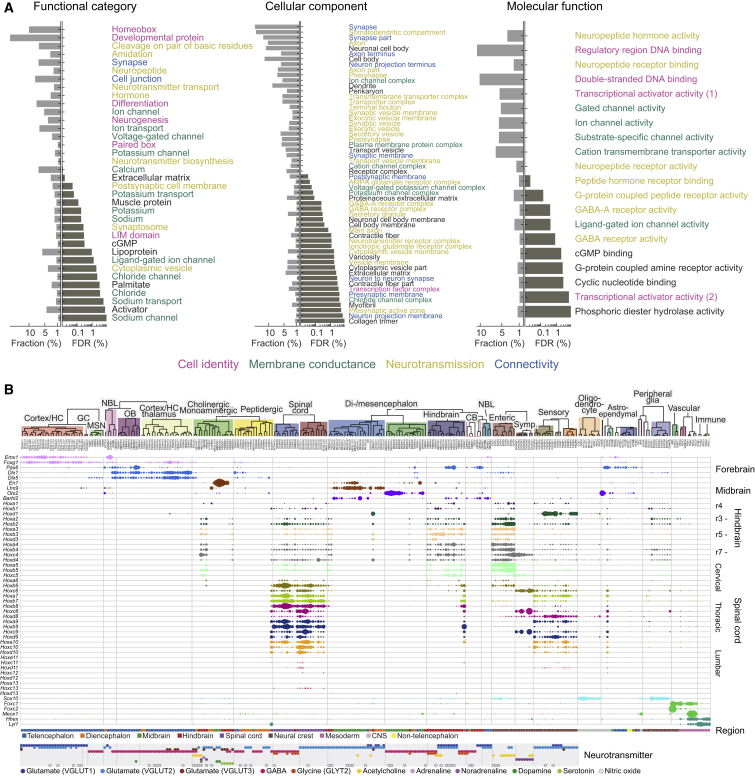
Drivers of Cellular Diversity (A) Gene ontology analysis of the most highly enriched genes in CNS neuronal clusters. Each panel shows the significantly (false discovery rate [FDR] < 10%) enriched terms ranked by FDR. Bars show the percentage of all genes (belonging to each term) that were enriched and the FDR. Colors indicate major categories of terms, as indicated below the figure. (B) Gene expression of developmental patterning transcription factors is shown along the cell-type taxonomy. Each row represents one transcription factor, and columns represent clusters. Circles represent mean expression values proportional to area. Genes are sorted according to their expression pattern, with Hox genes sorted rostrocaudally. Labels on the right indicate the approximate anatomical extent of the expression of corresponding *Hox* genes. See also Figures S4, S6, and S7.

Expanding the analysis to all genes, we note that the dendrogram and taxonomy (Figure 1C and Table S5) reflect systematic patterns of shared and unique gene expression. In order to understand what drives neuronal diversity, we collected the top ten most highly enriched genes in each neuronal cell type, reflecting both high expression and high specificity. A gene set enrichment analysis against the gene ontology (Figure 7A) (Huang et al., 2009) pointed to four clear categories of genes: those that establish cell identity (e.g., transcription factors, developmental genes), membrane conductance (e.g., ion channels, calcium-binding proteins), neurotransmission (e.g., neurotransmitter synthesis enzymes, transporters, neuropeptides, and their receptors), and synaptic connectivity (e.g., synaptic and cell junction proteins). These findings point to the specific functions that differ between neuronal types (connectivity, electrophysiology, and neurotransmission) and to the underlying regulatory machinery (transcription factors).

**Figure S7:**
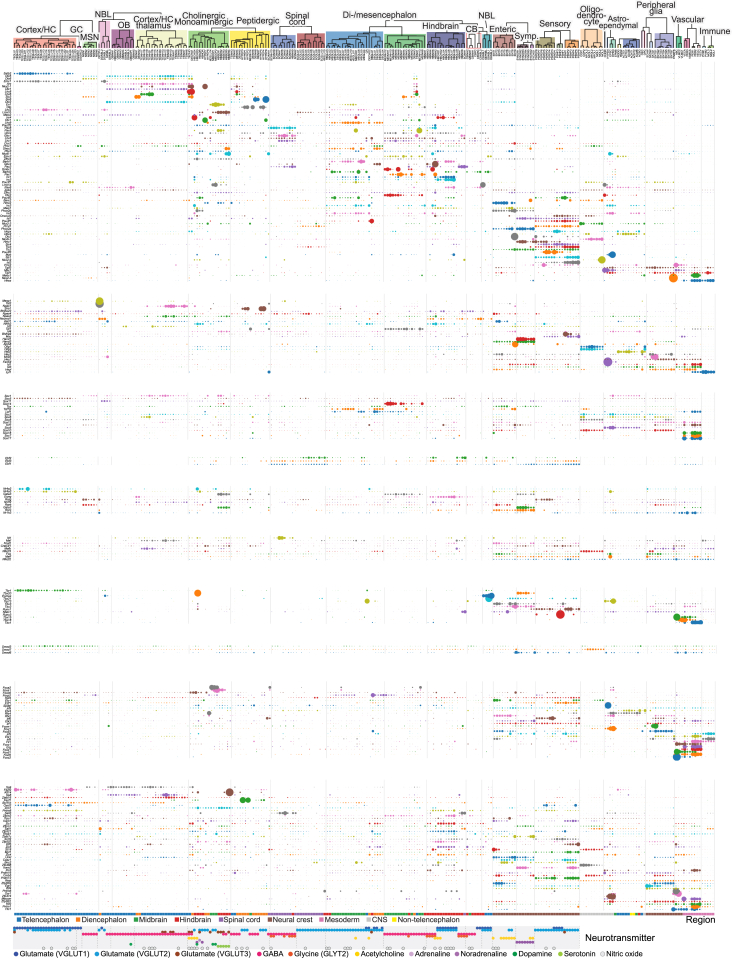
Expression Patterns of Transcription Factors, Related to Figure 7 Each section shows the expression of the indicated family of transcription factors, omitting genes that showed uniform or no expression. Circle areas are shown proportional to average gene expression in each cluster, normalized by row.

The gene family that best distinguished CNS neuron classes was homeodomain transcription factors (Figure S7), consistent with an important role in specifying and maintaining neuronal cell types. Many homeodomain transcription factors are involved in dorsoventral and anteroposterior patterning (as well as the specification of, for example, the neural crest). Although patterning takes place during embryogenesis, we reasoned that significant traces of patterning gene expression might remain and could explain the observation that the nervous system was molecularly organized according to developmental origin. In agreement with this prediction, we found that *Hox* genes were expressed in cell types derived from the hindbrain, spinal cord, and the PNS (Figure 7B). For example, spinal cord cell types expressed *Hoxa1* (rhombomere 1) through *Hoxd10* (thoracic bordering on lumbar), with additional expression of lumbar *Hox* genes in some cell types. Enteric neurons and glia of the small intestine both expressed a *Hox* code consistent with a major vagal and minor thoracic origin of these cells. Sensory and sympathetic neurons, as well as satellite glia, expressed *Hox* genes from all rostrocaudal levels (lumbar cells were not analyzed in the sympathetic nervous system). However, curiously, sympathetic neurons showed highly preferential expression from the *HoxC* cluster only. This is reminiscent of the role of the *HoxD* cluster during digit formation (Deschamps, 2008) and suggests that the *HoxC* cluster may be involved in the specification of distinct sympathetic cell types along some spatial axis.

*Hox* genes are not expressed in the forebrain and midbrain. Nevertheless, as in the hindbrain and spinal cord, forebrain cell types retained patterning gene expression. For example, the forebrain patterning gene *Foxg1* was found in all forebrain neurons, as well as in telencephalon-specific astrocytes. Dorsoventral patterning was also preserved: the dorsal gene *Emx1* was expressed in cortical, hippocampal, and striatal projection neurons, whereas ventral *Dlx1* and *Dlx5* were found mainly in inhibitory neurons of the same tissues.

## Discussion

We have described the molecular architecture of the mammalian nervous system based on a systematic survey using scRNA-seq.

Although we present a comprehensive analysis, our data have several limitations. First, there were technical and experimental limitations as detailed above, including doublets, sex-specific gene expression, and low-quality cells. Second, we sampled only a little more than half a million cells across the nervous system, and deeper sampling is likely to reveal additional structure that was obscured in the present study. Similarly, we used relatively shallow sequencing, and deeper sequencing using more sensitive RNA-seq methods is likely to resolve more subtypes. Third, some cell types may have been lost to differential survival or size selection biases (for example, Purkinje cells were likely undersampled here due to their size). Fourth, we have performed a very conservative clustering, designed to reveal clearly distinct major cell types, but did not analyze the substantial remaining heterogeneity within clusters. Finally, we have described only molecular cell types, but the task of linking molecular properties to functional, anatomical, morphological, and electrophysiological properties remains.

We suggest that the diversity of gene expression patterns in the nervous system can be understood through three major principles.

First, major classes of cells—e.g., neurons, astrocytes, ependymal cells, oligodendrocytes, vascular cells, and immune cells—are distinguished by large sets of class-specific genes that implement the specific function of each class of cells. For example, neurons share an extensive gene program involving synaptic, cytoskeletal, and ion channel genes, while oligodendrocytes express gene programs required for generating myelin. Multiple levels of hierarchical subdivision exist within these classes; for example, within neurons, neurotransmitter phenotype showed a modular and highly regulated pattern of expression.

Second, some—but only some—cell classes show area-specific patterns of gene expression that likely reflect their developmental history. This trend was strongest among neurons, astrocytes, and ependymal cells; by contrast, oligodendrocytes, vascular cells, and immune cells exhibited similar gene expression patterns across brain regions. The territories defining these gene expression domains corresponded closely to those marked out by embryonic morphogens, and spatial differences in adult expression patterns correlated with persistent expression of developmental transcription factors. This suggests that transcription factor networks induced in early development by local morphogens result in heritable regulatory states, which in turn are relayed into the diversification of terminal neuronal and astrocytic types specific to each brain region.

The fact that oligodendrocytes did not show similar spatial patterns—despite being derived from the same initially patterned neural tube as neurons and astocytes—reveals a loss of regional patterning in the oligodendrocyte lineage, presumably because region-specific patterning is transient and not converted to permanent states in these lineages. Alternatively—as suggested by the fact that OPCs aligned molecularly with neural-crest-derived glia—the transformation to a neural-crest-like state may involve an endogenous and naturally occurring cellular reprogramming analogous to the reprogramming of cells by overexpression of transcription factors.

Third, a secondary diversification, more graded and less region specific, results from interaction with the local environment and likely reflects inducible gene regulatory networks that respond in graded and transient fashion to local molecular cues. This was observed most clearly in the oligodendrocyte lineage but likely occurs to some extent in all lineages.

It remains unclear why regional diversity is so important in neurons, and to some extent, astrocytes, but not in oligodendrocytes. Among CNS neurons, we found that four main categories of genes drive neuronal diversity: those involved in cellular identity (transcription factors), connectivity (synaptic proteins, junction proteins), neurotransmission (neurotransmitters, neuropeptides), and membrane conductance (ion channels, calcium-binding proteins, solute carriers). But synaptic connectivity, neurotransmission, and membrane conductance are uniquely neuronal properties, and their diversity between regions is consistent with the diverse computational roles of each neuronal circuit. Conversely, the relative homogeneity of oligodendrocytes points to a common function, myelination, across all regions. The intermediate behavior of astrocytes is therefore consistent with the emerging view that they are not simply support cells, but they play an active role in computational processing (Henneberger et al., 2010).

In summary, we provide a resource and an initial analysis revealing key principles of the molecular diversity and composition of the mammalian nervous system. The atlas can be used to identify genes and gene combinations unique to specific cell types, which in turn can be used to genetically target cells for visualization, ablation, optogenetic manipulation, gene targeting, and more. The atlas will also help us understand the function of specific genes—for example, those implicated in disease (Skene et al., 2018). This can lead to actionable hypotheses on the mechanism of disease, as well as identifying the relevant cell types to generate mouse models of human disease.

## STAR★Methods

### Key Resources Table

**Table:** 

REAGENT or RESOURCE	SOURCE	IDENTIFIER
**Chemicals, Peptides, and Recombinant Proteins**		

TripLE Express Enzyme	Life Technologies	Cat# 12605036
Collagenase/Dispase	Roche	Cat# 10269638001
Neurobasal-A Medium	Life Technologies	Cat# 10888022
L-Glutamine	GIBCO	Cat# 25030081
B27	GIBCO	Cat# 1750400
Penicillin-Streptomycin	Sigma	Cat# P4333
OptiPrep Density Gradient Medium	Sigma	Cat# D1556
Liberase TH	Roche	Cat# 05401135001
ProLong Diamond Antifade Mountant	Thermo Fisher Scientific	Cat# P36961

**Critical Commercial Assays**		

Papain Dissociation System	Worthington	Cat# LK003163
Chromium Single Cell 3′ Library Kit v1	10X Genomics	Cat# 120230
Chromium Single Cell 3′ Gel Bead Kit v1	10X Genomics	Cat# 120231
Chromium Single Cell 3′ Chip Kit v1	10X Genomics	Cat# 120232
Chromium Single Cell 3′ Library & Gel Bead Kit v2	10X Genomics	Cat# 120237
Chromium Single Cell 3′ Chip Kit v2	10X Genomics	Cat# 120236
RNAScope Mulitplex Fluorescent Reagent Kit	Advanced Cell Diagnostics	Cat# 320850

**Deposited Data**		

Raw sequence data	NCBI SRA	Acc# SRP135960
Companion Wiki	Linnarsson lab	http://mousebrain.org
Loom Interactive Viewer	Linnarsson lab	http://loompy.org

**Experimental Models: Organisms/Strains**		

Mouse: CD-1	Charles River	Cat# 022
Mouse: Swiss	Janvier Labs	N/A
Wnt1-Cre:R26Tomato (C57Bl6J background)	Madisen et al., 2010	N/A
Vgat-Cre:tdTomato	Ogiwara et al., 2013	N/A

**Oligonucleotides**		

FISH probe for: *Mfge8*	Advanced Cell Diagostics	Cat# 408771
FISH probe for: *Agt*	Advanced Cell Diagostics	Cat# 426941-C2
FISH probe for: *Aqp4*	Advanced Cell Diagostics	Cat# 417161-C3
FISH probe for: *Slc6a9*	Advanced Cell Diagostics	Cat# 525151
FISH probe for: *Slc6a11*	Advanced Cell Diagostics	Cat# 492661-C3
FISH probe for: *Islr*	Advanced Cell Diagostics	Cat# 450041
FISH probe for: *Gdf10*	Advanced Cell Diagostics	Cat# 320269-C2

**Software and Algorithms**		

Cell Ranger Analysis Pipeline	10X Genomics	http://10xgenomics.com/
Cytograph	This paper	https://github.com/linnarsson-lab/cytograph
Adolescent Mouse	This paper	https://github.com/linnarsson-lab/adolescent-mouse
MATLAB Version 2016B	MathWorks	https://www.mathworks.com

### Contact for Reagent and Resource Sharing

Further information and requests for reagents and resources should be directed to and will be fulfilled by the Lead Contact, Sten Linnarsson (sten.linnarsson@ki.se).

### Experimental Model and Subject Details

#### Mice

Table S1 details the animals used per experiment. In summary, male and female mice were postnatal ages P12-30, as well as 6 and 8 weeks old. We mainly used wild-type outbred strains CD-1 (Charles River) and Swiss (Janvier). *Wnt1-Cre:R26Tomato* (C57Bl6J background) (Danielian et al., 1998, Madisen et al., 2010) were used to isolate peripheral and enteric nervous system, and *Vgat-Cre:tdTomato* (heterozygous for *Cre* and homozygous for *tdTomato*; mixed CD-1, C57BL/6J background) (Ogiwara et al., 2013) to isolate inhibitory neurons (vesicular GABA transporter, *Slc32a1*). Mice were housed under standard conditions and provided chow and water *ad libitum*. All experimental procedures followed the guidelines and recommendations of Swedish animal protection legislation and were approved by the local ethical committee for experiments on laboratory animals (Stockholms Norra Djurförsöksetiska nämnd, Sweden).

### Method Details

#### Single-cell dissociation

##### Brain

Single cell suspensions of all brain regions, i.e., all regions except spinal cord, sympathetic and enteric nervous system as well as dorsal root ganglia, were prepared as described previously (Hochgerner et al., 2018). Briefly, mice were sacrificed with an overdose of isoflurane, followed by transcardial perfusion with artificial cerebrospinal fluid (aCSF, in mM: 87 NaCl, 2.5 KCl, 1.25 NaH_2_PO_4_, 26 NaHCO_3_, 75 sucrose, 20 glucose, 1 CaCl_2_, 7 MgSO_4_). The brain was removed, 300μm vibratome sections collected and the regions of interest microdissected under a stereo microscope with a cooled platform. The pieces were dissociated using the Worthington Papain kit, with 25-35 min enzymatic digestion, as needed, followed by manual trituration using fire polished Pasteur pipettes and filtering through a 30μm aCSF-equilibrated cell strainer (CellTrics, Sysmex). Cells were then pelleted at 200 g, 5 min, supernatant carefully removed, and resuspended in a minimal volume aCSF. After manually counting cell concentration using a Burker chamber, suspensions were further diluted to desired concentrations. For improved cell viability, the composition of aCSF was altered (NMDG-HEPES) for experiments using P60 mice (see Table S1) (in mM): 93 NMDG, 2.5 KCl, 1.2 NaH2PO4, 30 NaHCO3, 20 HEPES, 25 glucose, 5 sodium ascorbate, 2 thiourea, 3 sodium pyruvate, 10 MgSO4, 0.5 CaCl2; adjusted to pH 7.4. To reduce debris when dissociating strongly myelinated regions, after filtering, the suspension was diluted in a large volume (15ml total) aCSF, followed by centrifugation, as above. Importantly, aCSF equilibrated in 95% O_2_ 5% CO_2_ was used in all steps, and cells were kept on ice or at 4°C at all times except for enzymatic digestion.

##### Spinal cord, sympathetic and dorsal root ganglia

CD-1 mice (DRG and spinal cord) or *Wnt1-Cre:R26RTomato* mice (sympathetic) were sacrificed and tissues of interest collected in freshly oxygenated, ice cold aCSF (see above). Sympathetic (SG) and dorsal root ganglia (DRG) were dissected and dissociated as described before (Furlan et al., 2016), with minor modifications. Briefly, following dissection (DRG: ∼30 ganglia collected in total from Cervical_1_-Lumbar_6_; SG: thoracic_1-12_ and stellate), the ganglia got transferred into a 3cm plastic dish with 2.7ml of pre heated (37°C) digestion solution (400μl TrypLE Express (Life Technologies), 2000μl Papain (Worthington; 25U/ml in aCSF), 100μl DNase I (Worthington; 1mM in aCSF) and 200μl Collagenase/Dispase (Roche; 20mg/ml in CS)). Non-ganglia tissue was removed from the ganglia. After 30 min incubation at 37°C, ganglia were triturated with 0.5% BSA-coated glass Pasteur pipette (flamed to 70% of original opening). DRG were also carefully ripped open by using fine forceps to make cells more accessible for the enzymes. This procedure was repeated every 20-30 min using Pasteur pipettes with decreasing diameter appropriate to the dissociation state. Depending on the dissociation progress 50μl of Collagenase/Dispase (20mg/ml) and 100μl of TrypLE solution was added.

Dissociation of the spinal cord followed the procedure described in Häring et al., 2018. In short, following the isolation of gray matter (from cervical to sacral levels), the tissue was transferred into a 3cm plastic dish with 2.5ml of pre heated (37°C) digestion solution (300μl TrypLE Express (Life Technologies), 2000μl Papain (Worthington; 25U/ml in aCSF), 100μl DNase I (Worthington; 1mM in aCSF) and 100μl aCSF. Meninges were removed and the gray matter cut into pieces 1-2mm^2^. After 30 min incubation at 37°C, pieces were triturated with the first Pasteur pipette (see above). This procedure was repeated every 20min using Pasteur pipettes with decreasing diameter appropriate to the dissociation state. Depending on the progress of spinal cord dissociation, 100μl of TrypLE solution was added.

As soon as all ganglion or spinal cord pieces were dissociated (DRG, SG: ∼1.5-2h; Spinal Cord: 45-60min), the cell suspensions were filtered using a 40μm cell strainer (FALCON) and collected in a 15ml plastic tube. The digestion solution was diluted with 3ml aCSF and centrifuged at 100 g for 4min at 4°C. The supernatant was removed and the pellet resuspended in 0.5ml aSCF and 0.5ml complete Neurobasal medium (Neurobasal-A supplemented with L-Glutamine, B27 (all GIBCO) and Penicillin/Steptamycin (Sigma)). The cell suspension was carefully transferred with a Pasteur pipette and layered on top of an Optiprep gradient: 90μl (DRG) or 80μl (SG) Optiprep Density Solution (Sigma) in 455μl aCSF and 455μl complete Neurobasal; and for spinal cord 170μl of Optiprep in 915μl aCSF and 915μl complete Neurobasal. The gradient was centrifuged at 100 g for 10min at 4°C, the supernatant removed until only 100μl remained and 10μl DNaseI added to avoid cell clumping.

##### Enteric nervous system

*Wnt1-Cre;R26RTomato* mice were killed by cervical dislocation followed by dissection of small intestine. During all steps the tissue was kept in aCSF (in mM: 118 NaCl, 4.6 KCl, 1.3 NaH_2_PO_4_, 25 NaHCO_3_, 20 glucose, 7 mM CaCl_2_ and MgSO_4_) equilibrated in 95% O2 5% CO2 for 30 min before use and held on ice. The small intestines of male and female (P21) mice were cut in 5cm pieces and flushed clean with ice-cold aCSF using a blunt 20G needle attached to a 20ml syringe. The mesentery was removed, the pieces opened lengthwise along the mesenteric border and pinned with the mucosa side down on a Sylgaard (Dow Corning) covered dissection dish. The outer smooth muscle layers, containing the myenteric plexus were peeled off from the submucosa using forceps. The tissue was digested in 1,5 mg/ml Liberase (Grundmann et al., 2015), 0.1 mg/ml DNaseI and 1xAntibiotic-Antimycotic (ThermoFisher) in aCSF at 37°C for 1h, with shaking of the tube every 15 min. The cells were gathered by centrifugation at 356 g for 5min followed by incubation in TrypLE for 30 min. The suspension was washed in aCSF, centrifuged at 356 g for 5 min and resuspended in aCSF, 1% BSA. After manual trituration using BSA-coated fire-polished Pasteur pipettes with decreasing opening size, the single cell suspension was filtered through 70μm filter (Miltenyi Biotec) and cleaned of debris by centrifugation through 1 mL FBS at 800 g for 10min. The cells were resuspended in oxygenated aCSF, 1%BSA and filtered through a 30μm filter (Miltenyi Biotec). Tom+ cells were FAC sorted on a BD FACSAria II and collected in ice-old aCSF.

#### Single-Cell RNA-seq

The majority of sampling was carried out with 10X Genomics Chromium Single Cell Kit Version 1, although part of the hindbrain sampling was done in Version 2 (Table S1). Suspensions were prepared as described above and diluted in aCSF, to concentrations between 300-1000 cells/μl (listed in Table S1), and added to 10x Chromium RT mix to achieve loading target numbers between 2500-8000 (V1 kit) or 7000-10,000 (V2 kit), as indicated. For downstream cDNA synthesis (12-14 PCR cycles), library preparation, and sequencing, we followed the manufacturer’s instructions.

#### RNA-ISH

CD-1 mice (Charles River) were killed with an overdose of isoflurane and transcardially perfused with aCSF (see Single Cell Dissociation, Brain). Brains were dissected out, snap frozen in OCT on a bath of isopentane with dry ice and stored at –80°C. Fresh frozen sagittal whole-brain sections (including the olfactory bulb, SVZ, hippocampus and cerebellum) of 10 μm thickness were cryosectioned and stored at –80°C. Sections were thawed just prior to staining and fixed with 4% PFA for 15 min followed by rinsing in PBS. RNAscope *in situ* hybridizations were performed according to the manufacturer’s instructions, using the RNAscope Multiplex Fluorescent kit (Advanced Cell Diagnostics) for fresh frozen tissue, as previously described (Hochgerner et al., 2018). A 10 min treatment in SDS (4% in 200 mM sodium borate) was added in the protocol after the Protease IV incubation. Following probes with suitable combinations were used (indicated with gene target name for mouse and respective channel, all Advanced Cell Diagnostics): *Mfge8* (Ch1), *Agt* (Ch2), *Aqp4* (Ch3), *Slc6a9* (Ch1), *Slc6a11* (Ch3), *Islr* (Ch1) and *Gdf10* (Ch2). All sections were mounted with Prolong Diamond Antifade Mountant (Thermo Fisher Scientific). Imaging was carried out on a Nikon Ti-E epifluorescence microscope at 10X magnification.

### Quantification and Statistical Analysis

#### Cytograph pipeline

Chromium samples were sequenced, typically one sample per lane, per the manufacturer’s instructions with one 98 bp read located near the 3ʹ end of the mRNA. Illumina runs were demultiplexed, aligned to the genome and mRNA molecules were counted using the 10X Genomics *cellranger* pipeline.

Each raw Chromium sample was manually inspected after sequencing. Samples that showed no obvious structure in their t-SNE plots (generated automatically by the Chromium cellranger pipeline) were excluded from further analysis. The complete list of input samples is given in Table S1.

All subsequent analyses were automated in the *cytograph* library and *adolescent-mouse* pipeline, freely available as open source. *Cytograph* evokes both the fact that our cell type clustering and visualizations are graph-based, and the fact that the pipeline itself is organized as a directed acyclic graph.

Our pipeline is based on Luigi (Spotify), a Python-based software that orchestrates a set of tasks with dependencies. Each task takes zero or more input files, and generates exactly one output file. Luigi automatically determines which outputs are missing, and the order in which tasks have to be executed to generate them. It can also allocate independent tasks in parallel, to increase throughput.

Cells with less than 600 detected molecules (UMIs), or less than 1.2-fold molecule to gene ratio, were marked invalid. Genes detected in fewer than 20 cells or more than 60% of all cells were marked invalid. These filters were applied separately to each input file.

##### Preliminary exploratory analysis

In preliminary analyses, we explored a large number of approaches for dimensionality reduction, manifold learning, clustering and differential expression analysis methods, in order to get a deep preliminary understanding of the dataset.

For normalization and noise reduction, we tried simple things like mean-centering, normalization to a common molecule count, standardization (division by the standard deviation) and log transformation; we also explored MAGIC (a method that imputes expression based on neighbors in the KNN graph) and diffusion maps.

For manifold learning, we projected the high-dimensional dataset either to a graph (e.g., of *k* nearest neighbors KNN, and variants such as mutual nearest neighbors) or to two or three dimensions (using PCA, t-SNE, SFDP). We also combined these approaches, first projecting to a graph, then calculating distances on the graph (e.g., Jaccard distance, or multiscale KNN distance; see below), then using those distances to project to 2D space using graph-t-SNE (gt-SNE; see below).

For clustering, we explored standard methods such as K-means (and iterative K-means) in PCA space, as well as graph-based algorithms (Louvain community detection) and density-based algorithms in 2D or 3D projections (e.g., DBSCAN, HDBSCAN).

The final algorithm choices below reflect what we learned in this exploratory phase.

##### Preliminary clustering and classification

We extensively mined clusters obtained in preliminary analyses and found that they largely corresponded to known and putative cell types, broadly consistent with previous data. Some clusters were also clearly derived from doublets, expressing contradictory markers e.g., from neurons and vascular cells.

With any type of clustering the choice of feature space is crucial. For preliminary clustering, we used genes informative across the entire set of cells, projected by PCA. This would be expected to be suitable for finding major cell types, but would not be optimal for finding finer subdivisions among cells of the same kind (e.g., interneurons in a dataset containing both neurons, vascular cells and glia). For example, running Louvain clustering on the full dataset resulted in only 44 clusters, compared to the 265 found by the multi-level, iterative approach described below.

We decided to first split cells by major class. In order to split the data, and to reject many doublets, we trained a classifier to automatically detect the major class of each single cell, as well as classes representing doublets. We first manually annotated clusters to indicate major classes of cells: *Neurons, Oligodendrocytes, Astrocytes, Bergman glia, Olfactory ensheathing cells, Satellite glia, Schwann cells, Ependymal, Choroid, Immune,* and *Vascular.* For some of these classes, we distinguished proliferating cells (e.g., *Cycling oligodendrocytes*, i.e., OPCs). We also manually identified clusters that were clearly doublets between these major classes (e.g., *Vascular-Neurons*) as well as clusters that were of poor quality.

We then trained a support vector classifier to discriminate all of these labels, using the training set of preliminary clusters manually annotated with class labels. We sampled 100 cells per cluster and used 80% of this dataset to optimize the classifier, and the remaining 20% to assess performance. On average, the classification accuracy was 93% for non-cycling cells. The precision and recall for neurons was 93% and 99%, respectively. That is, 99% of all neurons were classified correctly, and 93% of all cells classified as neurons were actually neurons. The classifier struggled to distinguish cycling cells, presumably because they shared most gene expression with their non-cycling counterparts. For this reason, we always pooled cycling and non-cycling cells after classification. The table below shows the accuracy for all major classes of interest:

**Table:** 

	Precision	Recall
astrocyte	87%	96%
astrocyte, cycling	59%	38%
bergmann-glia	100%	97%
blood	77%	65%
ependymal	98%	97%
immune	96%	98%
neurons	93%	99%
neurons, cycling	63%	54%
oec	100%	95%
oligos	91%	97%
oligos, cycling	39%	19%
satellite-glia	90%	95%
satellite-glia, cycling	91%	88%
schwann	100%	100%
choroid	100%	80%
vascular	87%	97%
vascular, cycling	100%	25%

**average (non-cycling)**	**93%**	**93%**

We used this classifier to individually assess the class identity of each cell in each dataset, and to pool cells by major class into new files (with neurons further separated by tissue).

##### Removing doublets

We expected about 2% of all cells to be doublets. Preliminary exploratory analysis (including by generating simulated doublets) showed that most doublets would either form separate clusters, or would tend to end up at the fringes of other clusters (in graph embeddings, and in t-SNE). To eliminate many doublets, we (1) removed clusters classified with ambiguous labels; (2) removed cells classified with a different label from the majority of cells in its clusters; (3) removed outliers when clustering, typically on the fringes of clusters in t-SNE space.

##### Level 1 analysis

We pooled samples by tissue and performed manifold learning, clustering, classification, gene enrichment, and marker gene detection (see below for details on these procedures).

##### Level 2 analysis

We split cells by major class according to the class assignment probability. For each cluster at level 1, we removed cells with conflicting classification (i.e., cells classified as *Neuron* in a cluster where the majority of cells are classified as *Vascular*). We performed the same analysis steps as for Level 1.

##### Level 3 analysis (neurons)

Because of the way we had dissected the brain, we would expect some clusters to appear in multiple tissues. For example, our olfactory sample included the anterior-most part of the cortex and underlying tissue (intended to cover the anterior olfactory nucleus), and could overlap with the cortex samples. In order to allow clusters to merge across such boundaries, and in order to improve resolution in clustering, we pooled cells in broader categories, and split them by (mostly) neurotransmitter, as follows:

**Table:** 

Region	Class
spinal cord	GABAergic, glycinergic
spinal cord	glutamatergic
pns	all
hypothalamus	peptidergic
hindbrain	GABAergic, glycinergic
hindbrain	glutamatergic
whole brain	neuroblasts
forebrain	GABAergic
forebrain	glutamatergic
di- and mesencephalon	GABAergic
di- and mesencephalon	glutamatergic
whole brain	granule cells
whole brain	cholinergic and monoaminergic
striatum	medium spiny neurons

Note: we pooled granule cells of the dentate gyrus and the cerebellum not because we think they are related (they are not), but because they are both extremely abundant and tended to skew manifold learning when included with other cells.

##### Level 4 analysis

Despite our efforts, at level 3 there remained still some clusters that were suspected doublets, as well as over-split clusters that lacked clearly defining gene expression differences. We therefore manually curated all clusters, merging some and eliminating others. We then recomputed the manifolds, but did not recluster.

##### Level 5 analysis

To create the final consolidated dataset, we extensively annotated and named each cluster (Table S3). We pooled all cells into a single file along with all metadata and annotations, and performed gene enrichment analysis and marker gene set discovery on this dataset. The level 5 analysis was the basis for all downstream analysis.

##### Level 6 analysis

Finally, level 6 is identical to level 5, but organized into subsets according to the taxonomy (Figure S3). This provides gene enrichment analysis and marker gene set discovery, individually for each taxon.

#### Manifold learning

Each individual cell can be viewed as a point in a high-dimensional space, with coordinates given by the expression of every gene. This space would have about 27,000 dimensions, one per gene. In principle, cell types can be viewed as high-density regions in this space, and clustering methods can be used to find them.

In some sense, cells reside on a low-dimensional manifold in the high-dimensional gene expression space. However, the high dimensionality and sparseness of this space creates the “curse of dimensionality,” where distance measures essentially stop making sense. A second issue concerns measurement noise, with generally low counts and large numbers of dropouts (false negatives). Both of these issues can be mitigated by (1) selecting a reduced set of informative genes and (2) linearly projecting the data to a transformed space where each coordinate corresponds to many co-regulated genes. The most effective way of selecting informative genes, would be to select them relative to known classes. We therefore developed a staged procedure to learn the manifold.

We first selected 1000 informative genes by fitting a support-vector regression to the coefficient of variation (CV) as a function of the mean, and selecting genes having the greatest offset from the fitted curve; this would correspond to genes with higher-than-expected variance. We excluded the sex-specific genes *Xist* and Tsix. We normalized each cell to a sum of 5,000 molecules (UMIs), then log-transformed and subtracted the mean (per gene).

We then used principal component analysis (PCA) to both reduce noise and to reduce the gene expression space further. Dropping non-significant principal components (Kolmogorov-Smirnov test, p < 0.05) reduced the space to a few tens of dimensions (typically about forty).

Given a reduced and denoised dataset, we next sought to learn the shape of the manifold of cells (that is, the underlying lower-dimensional gene expression space on which cells are preferentially located). Examining the PCA revealed that the manifold consisted of feather-like, elongated structures, extending variously into the different principal components. We found that the manifold was structured at many levels, ranging from broadly different classes of cells, individual cell types, to more subtle sub-types or states.

We constructed a balanced mutual *k* nearest-neighbor (KNN) graph with *k* = 100 using Euclidean distance in the space of significant components. We allowed a maximum of 200 incoming edges to each cell and then dropped all non-mutual edges. We performed Jaccard multilevel community clustering on this graph to define a preliminary set of cell types/states.

Given this preliminary clustering, we were able to select an even more informative set of 500 genes, by calculating an enrichment score (see below) for each cluster, and selecting the ten most highly enriched genes for each cluster.

Next, we repeated the procedure (PCA, mutual KNN, clustering) with modifications as follows. First, for computing the PCA transform, we limited the number of cells from the largest clusters to contribute max 20% of the total cells (to avoid skewing the PCA toward dominant cell types; note that we still kept all cells in the dataset, only masking those cells when computing the PCA transformation matrix). Second, we computed a balanced KNN as before but we assigned weights $w_{i,j}=1/k^{\alpha }$, where *k* is the rank of *j* among the neighbors of *i* and *a* is a power that sets the scale of the weights. Large values of a will emphasize local neighborhoods, whereas smaller values will emphasize global structure, but in both cases, both local and global structures are accounted for. For practical purposes, we calculated the multiscale graph only up to *k* = 100 (beyond which the edge weights are vanishing), and we used *a* = 1. Using a fixed maximal k also ensured that the algorithm remained linear in the number of cells. We call this a multiscale KNN, and stored both the KNN and the mutual KNN for use in further clustering and visualization (available as column graphs named KNN and MKNN in the Loom files).

We projected the multiscale KNN graph to two dimensions using a modified t-SNE algorithm we call graph t-SNE (gt-SNE). In contrast to standard t-SNE, which is based on distance measures, we directly projected the multiscale KNN, which is based on multiscale weighted ranks. We achieved this by replacing the distance matrix *P* in regular t-SNE with the distance matrix of the weighted multiscale KNN graph. The result was a more accurate projection of the graph itself, with more compact and well-defined neighborhoods. We stored the gt-SNE embedding as column attributes _X and _Y in the Loom files.

#### Clustering

Finally, we performed clustering on the multiscale KNN graph. We used Louvain multilevel community clustering (Blondel et al., 2008). However, modularity-based graph clustering suffers a well-known resolution limit (Fortunato and Barthélemy, 2007), failing to find small clusters even when they are perfectly unambiguously defined. Some variants (so called resolution limit-free algorithms) can be tuned to detect smaller clusters, but at the expense of breaking up large clusters. To circumvent this issue, we exploited the fact that we had both a graph, and an embedding of the graph in two dimensions. We first used Louvain clustering on the graph to find most clusters, and then isolated and re-clustered each cluster using DBSCAN in the low-dimensional space. We call this approach “Polished Louvain.”

In more detail, we first performed Louvain community detection on the MKNN graph, with resolution set to 1.0 (except for level 3 where we used 0.6 for astrocytes, 0.35 for sensory neurons and 0.6 for granule cells).

We marked cells as outliers if they (1) belonged to clusters with less than ten cells; or (2) were marked as outliers by DBSCAN (on the 2D embedding) with å set to the 80^th^ percentile of the distance to the *k*^*th*^ nearest neighbor and min_samples = 10; or (3) if more than 80% of the cell’s nearest neighbors belonged to a different cluster.

Next, we isolated each cluster and considered it for further splitting, in the 2D space of the gt-SNE embedding. We centered it using PCA and standardized it by subtracting the mean and dividing by the standard deviation. We marked the cluster for splitting if it now showed three or more outliers based on the median absolute deviation (MAD) with threshold 3.5. We also marked the cluster for splitting if more than 5% of the cells (or 25 cells, whichever is larger) were located at a distance greater than the 70^th^ percentile of the distance to the *k*^*th*^ nearest neighbor.

If a cluster was marked for splitting, we performed DBSCAN on that cluster with å set to the 70^th^ percentile of the distance to the *k*^*th*^ nearest neighbor and min_samples = 5% of the cells (or 25 cells, whichever is larger).

Finally, we set the cluster label of each cell to the majority label of its ten nearest neighbors.

We stored cluster labels as column attribute Clusters in the Loom files (integer ranging from 0 to *n*). At level 5 and 6, cluster names are given by the column attribute ClusterName.

#### Gene enrichment

To aid interpretation of the data (and for gene selection, as noted above), we computed a set of genes enriched in each cluster. We computed an enrichment statistic $E_{i,j}$ for gene *i* and cluster *j*, as follows:

$${\text{E}}_{\text{i},\text{j}}=\left(\frac{{\text{f}}_{\text{i},\text{j}}+\varepsilon_{1}}{{\text{f}}_{\text{i},\bar{\text{j}}}+\varepsilon_{1}}\right)\left(\frac{{\text{ì}}_{\text{i},\text{j}}+\varepsilon_{2}}{{\text{ì}}_{\text{i},\bar{\text{j}}}+\varepsilon_{2}}\right).$$

where $f_{i,j}$ is the fraction of non-zero expression values in the cluster and $f_{i,\bar{j}}$ is the fraction of non-zero expression values for cells not in the cluster. Similarly, $\mu_{i,j}$ is the mean expression in the cluster and $\mu_{i,\bar{j}}$ is the mean expression for cells not in the cluster. Small constants $\varepsilon_{1}=0.1$ and $\varepsilon_{2}=0.01$ are added to prevent the enrichment score from going to infinity as the mean or non-zero fractions go to zero. Enrichment scores are available as matrix layer enrichment in the aggregated Loom files (named “…agg.loom”). We also computed an enrichment *q* value by shuffling the expression matrix, available as layer enrichment_q. To find genes enriched at a 10% false discovery rate, for example, simply select genes with *q* scores below 0.1.

#### Trinarization

It is often useful to estimate (for each cluster) if a gene is likely expressed, not expressed, or we are not sure. That is, we want to trinarize the raw expression data into calls of *expressed*, *not expressed*, and *indeterminate*. Here we used a Bayesian beta-binomial model to trinarize the raw data.

The model applies to a cluster of cells representing a putatively homogeneous population. In this cluster, we have measured gene expression in n cells, and for each cell we have either detected the gene, or not. Given detection in *k* out of *n* cells, we want to know the underlying population frequency of expression, *Θ*. The observed fraction of expressing cells can be expressed conditional on the number of cells and the population expression frequency. By providing a prior on *Θ*, we can derive the posterior distribution of *Θ* given the observed number of detections:

k|n,θ∼Binomial(θ,n)

θ∼Beta(a,b)

θ|n,k∼Beta(a+k,b+n−k)

where the *Beta* distribution is the conjugate prior to the *Binomial*, and as a consequence the posterior distribution is also *Beta*, and can be calculated simply by updating the parameters. Setting *a = b = 1* results in a non-informative uniform prior. Here, we used instead a weakly informative prior with *a = 1.5, b = 2*, which slightly favors not expressed and indeterminate calls.

Using this model to trinarize gene expression, we call a gene expressed when *P(Θ > f) > (1 - PEP)*, where f is the population fraction of cells expressing the gene, and PEP is the desired posterior error probability (also called local false discovery rate, or FDR). For example, with PEP = 0.05, there is less than 5% risk, given the observations, that the expressed call is wrong. Similarly, we call a gene not expressed when *P(Θ > f) < PEP*. For values between 1-PEP and PEP, we call the gene indeterminate. Note that PEP is applied individually to each gene (hence, ‘local FDR’) and the actual genome-wide FDR will be strictly equal to or lower than PEP.

The probability *P(Θ > f)* can be calculated as:

$$P\left(\theta >f\right)=1-\frac{B\left(f;a+k,\;b+n-k\right)\times B\left(a+k,b+n-k\right)\times \Gamma \left(\text{a}+\text{b}+\text{n}\right)}{\Gamma \left(\text{a}+\text{k}\right)\times \text{Ã}\left(\text{b}+\text{n}-\text{k}\right)}$$

B(z; a, b) is the regularized incomplete beta function, B(a,b) is the beta function, and Γ is the gamma function. The formula was derived by evaluating Probability[x > f, {x ∖[Distributed] BetaDistribution[a + k, b + n - k]}] in *Mathematica* (version 10, Wolfram Research Inc.).

Evaluating this function, for a given k and n (and hyperparameters f, a and b) yields a probability P, which we compare to the thresholds *1-PEP* and *PEP* to give the gene an expression call. We used *a = 1.5, b = 2, f = 0.2* and *PEP = 0.05* to make the calls in this paper, unless otherwise indicated. Thus a gene was considered expressed if it was estimated to be present in at least 20% of the cells with no more than 5% posterior error probability.

Note that the formula as written suffers from numerical instability when evaluated at finite precision. This problem can be avoided by using logarithms of the beta and gamma functions, and then exponentiating. See the source code of function p_half in file diff_exp.py for a complete, numerically stable implementation.

Trinarization scores are available in layer trinaries in the aggregated loom files.

#### Marker gene set discovery

Many, even most, of the cell types described in this paper were not previously associated with known makers. We therefore designed an algorithm to automatically propose marker sets for all clusters. Here, we define a marker gene set as a set of genes that are all expressed in a given cluster, but not all expressed in any other cluster. We used trinarization to judge if a gene is expressed or not in each cluster.

Given a cluster, we first selected the most highly enriched gene, which would often not be unique to that cluster, but highly selective for a small number of closely related clusters. Next, we added the most specific gene, based on trinarization with a PEP of 0.05. This gene was very often specific to a very small number of clusters, and using the first two genes together would often lead to fully specific marker combinations. However, sometimes adding more genes would be necessary.

We added genes one at a time by picking the most selective gene, *in combination with the previously selected genes*. When more than one gene was equally selective, we picked the one that was most highly enriched. We defined selectivity as the reciprocal of the number of clusters that would be assigned auto-annotation given the current gene set and the trinarization scores. That is, an annotation that would apply to *k* clusters would have selectivity 1/*k*. Adding more genes rapidly drove selectivity toward 1.

We generated gene sets in this manner for all clusters, with up to six genes per cluster. We also calculated the cumulative selectivity, specificity (difference between the posterior probability for the best cluster and that of the second-best cluster), and robustness (the posterior probability that all genes would be detected in the cluster, based on trinarization scores). We reported these statistics cumulatively for n = 1, 2, 3, 4, 5 and 6 genes. Generally, robustness drops as more genes are added, while selectivity increases. Specificity tends to increase as the gene set becomes more selective, but then decrease as it becomes less robust.

We note that marker gene sets are excellent candidates to use for experimentally identifying cell types, e.g., based on genetic or antibody labeling. Marker gene sets and associated statistics for all clusters are provided in the wiki, and in the Loom files under column attributes MarkerGenes, MarkerSelectivity, MarkerSpecificity and MarkerRobustness.

#### Neurotransmitter calling

To associate neuronal cell types with their neuotransmitters (Figures 1C, 7B, and S7 and Table S3), we trinarized the expression of genes coding for neurotransmitter transporters or enzymes crucial to their synthesis (Figure S6D, asterisks); combined with manual inspection of the expression on a single-cell level. Trinarization was carried out as described in the section above, with *f = 0.05* (present in at least 5% of cells) and posterior error probability (PEP) 0.05, except for *Nos1* where *f = 0.2*. Given a set of genes defining a neurotransmitter phenotype, we conservatively required all genes to pass the trinarization threshold. For glutamatergic neurons, we used the individual vesicular glutamate transporters (VGLUT1-3) separately.

Clusters CBPC, HYPEP3 and SCINH2 showed dual presence of GABA (inhibitory) and glutamate (excitatory) in the same cluster, but manual inspection indicated that this was artifactual (for example, contamination of Purkinje cells by granule cells in the cerebellum). Clusters OBINH4, SZNBL and DEINH6 showed no neurotransmitter by trinarization, but manual inspection indicated GABA. Clusters where ‘neurotransmitter genes’ were not expressed homogenously were also identified. All these manual interventions were documented in the “Comment” field of Table S3, in each loom file and in the wiki.

#### Dendrogram construction

All linkage and distance calculations were performed after $Log_{2}\left(x+1\right)$ transformation.

The starting point of the dendrogram construction was the 265 clusters. For each gene, we computed average expression, trinarization with *f* = 0.2, trinarization with *f* = 0.05 and enrichment score. For each cluster we also know the number of cells, annotations, tissue distribution and samples of origin.

We defined major classes of cell types based on prior knowledge: neurons, astroependymal, oligodendrocytes, vascular (without VLMC), immune cells and neural crest-like. For each class, we defined pan-enriched genes based on the trinarization 5% score. Each class (except neurons) was tested against neurons, to find all the genes where the fraction of clusters with trinarization score = 1 in the class was greater than the fraction of clusters with trinarization score > 0.9 among neurons.

In order to suppress batch effects (mainly due to ambient oligodenderocyte RNA in hindbrain and spinal cord samples), we collected the unique set of genes pan-enriched in the non-neuronal clusters, as well as a set of non-neuronal genes that we believe to have tendency to appear in floating RNA (*Trf, Plp1, Mog, Mobp, Mfge8, Mbp, Hbb-bs, H2-DMb2*) and a set of immediate early genes (*Fos, Jun, Junb, Egr1*). These genes were set to zero within the neuronal clusters to avoid any batch effect when clustering the neuronal clusters. We further removed sex specific genes (*Xist, Tsix, Eif2s3y, Ddx3y, Uty,* and *Kdm5d*) and immediate early genes Egr1 and Jun from all clusters.

We bounded the number of detected genes in each cluster to the top 5000 genes expressed, followed by scaling the total sum of each cluster profile to 10,000.

Next, we selected genes for linkage analysis: from each cluster select the top N = 28 enriched genes (based on pre-calculated enrichment score), perform initial clustering using linkage (Euclidean distance, Ward in MATLAB), and cut the tree based on distance criterion 50. This clustering aimed to capture the coarse structure of the hierarchy. For each of the resulting clusters, we calculated the enrichment score as the mean over the cluster divided by the total sum and selected the 1.5*N* top genes. These were added to the previously selected genes.

Finally, we built the dendrogram using linkage (correlation distance and Ward method).

##### Test for dendrogram stability

We tested the stability of the dendrogram structure while changing the number of genes selected for calculating the dendrogram. We selected *N* in the range 10-44. For each *N* we repeated the procedure above and stored the selected genes and dendrogram structure. We then examined all branches (junctions) of the reference tree (N = 28) and compared them to the corresponding branch in the test tree. We derived two stability criteria (1) branches with leafs below having 90% overlap in test compared to reference, (2) branches with exactly the same set of clusters and the same order of the leafs. For each branch we calculated the fraction of cases that either criteria (1) or (2) occurred. More than 65% of the 264 branches had probability of 1 and 94% had probability greater than 0.5 based on criterion (1). Based on the more stringent criterion (2) more than 50% had probability of 1 and about 85% greater than 0.5.

##### Testing for dendrogram without any gene exclusion

In the dendrogram construction described above we used several steps of exclusion genes either from all clusters or from the neuronal clusters in particular. This was done due to our observation of background levels of gene detection which seemed to be depend on very abundant cell types the dissected region (e.g., oligodendrocytes in hindbrain or enteric glia in the enteric nervous system). This is likely because of floating RNA coming from dead cells or doublets either with abundant cells or parts of broken cells. Still, due to the risk of misinterpreting the data we also constructed the dendrogram based on similar procedure but without any gene exclusion. The resulting dendrogram was not fundamentally changed from Figure 1C, but included a few key differences which we believe are technical artifacts. First, enteric neurons clustered together with the enteric glia probably due to fact that enteric glia were extremely abundant in the tissue. This created a big enough change that the other PNS neurons created a separate branch disconnected from the other neurons. Second, the olfactory bulb inhibitory neurons were placed next to the MSNs. This branch in turn was connected to a branch mainly containing neuroblasts. Finally, the OPC cluster was placed next to the SZNBL cluster probably because of strong cell-cycle signal.

#### Spatial correlation analysis

Our aim here is the try and map the gene expression profile at the cluster level to the mouse *in situ* hybridization atlas of the Allen Institute for Brain Research (http://mouse.brain-map.org) (Lein et al., 2007). The Allen Mouse Brain Atlas was summarized into a 200 μm voxel dataset, providing the gene expression profile (all genes) for each voxel. In this analysis we used simple correlation between the voxel gene expression (from *in situ* hybridization) and the cluster gene expression profile (from scRNaseq).

For each gene, the voxel data is a 67 × 41 × 58 (rows × columns × depth) array, giving an “energy” value representing the expression. In addition, for each voxel we know the anatomical annotation. The Allen Brain reference atlas is given at a finer resolution with voxels of 25μm (528 × 320 × 456). In order to achieve finer resolution and smoother images we used linear interpolation of the coarse (200μm) *in situ* data into the finer grid (25μm). For annotation we used the color code of the Allen reference atlas.

Since many genes have information only from sagittal sections of one hemisphere, we can neglect one hemisphere also from the genes that have coronal data. Coronal data is preferred since it has better sampling.

##### Procedure

First, we define the energy of any voxel outside the valid domain to −1. We define genes as high-quality (*in situ* data) when they satisfy: average voxel energy > 0.2 and more than 30 voxels higher than 5. This is calculated over the valid domain voxels. The thresholds were based on inspection of the mean versus CV, variance etc. (data not shown). Next, we normalized the voxel energy: for each gene, transform the energy by

$$\frac{\log_{2}\left(voxel\;energy\left(i,in\right)+1\right)-m}{s}$$

where $m=mean\left(\log_{2}\left(voxel\;energy\left(i,in\right)+1\right)\right)$; $s=std\left(\log_{2}\left(voxel\;energy\left(i,in\right)+1\right)\right)$ and in=voxelenergy(i,:)>0

We then loaded aggregate (mean per cluster) data for each cell type and selected the genes as described above for dendrogram construction analysis. We then intersected the selected genes from aggregate data and quality filter on energy voxel data. We calculated the correlation between each voxel and each cell-type, where voxel data was normalized as above and the aggregate data was normalized in a similar way (*(X-m)/s*) after $\log_{2}+1$ transform. Finally, we calculated the regional fold enrichment: for each cell-types take the top 100 pixels (across the whole brain) and calculate the fold-enrichment of the anatomical region IDs that are among them by normalizing to frequency within the 100 to the overall frequency of each region ID.

#### Image analysis for astrocyte markers

##### Analysis of RNAscope *in situ* hybridization images

We performed three sets of RNAscope stainings as described in Method Details; Staining (1) *Islr, Aqp4, Gdf10* (2) *Mfge8, Aqp4, Agt* (3) *Slc6a9, Slc6a11, Agt*. Full sagittal brain sections were scanned and stitched to a large image as described above. To quantify expression of the relevant markers (Figure 3F), we processed the images as follows. In order to allow overlap of spatial information from multiple images we aligned the three images using a set of 16 reference points. We manually registered these points using the DAPI channel as guide for the general anatomy. Images were transformed into the common coordinates using affine transform (MATLAB, “*fitgeotrans*” function) on the reference points and pixels outside the relevant domain were set to zero. The steps for image processing were as follows: (1) Enhance each channel between percentile 50 to 100. (2) Calculate background using “*adapttresh*” function MATLAB. (3) Subtract the background. (4) Re-enhance with gene specific parameter. (5) To obtain the RNAscope spots binary image, calculate extended maxima transform (“*imextendemax*” MATLAB), followed by fill holes (“*imfill*”) and remove all objects larger than 50 pixels (“*bwareaopen*”). (6) Spots were required to be inside the DAPI region. (7) Cell domains (as defined by DAPI) with greater than 2 spots were consider positive. Positions of positive cells for the genes mentioned is presented in Figure 3F.

##### Analysis of Allen Mouse Brain Atlas *in situ* images

In order to independently validate our findings about the spatial distribution of astrocytes (Figure S5C), we analyzed *in situ* hybridization images of marker genes obtained from the Allen Mouse Brain Atlas (http://mouse.brain-map.org) (Lein et al., 2007). First, we downloaded sagittal section images of the following marker genes: *Islr, Gdf10, Agt, Mfge8*. For overlay of these images and we used similar strategy as described above fro RNAscope. Reference points (16 points) were manually registered in each image. We used the “Expression” images and the following steps. (1) Transform to the common coordinates (MATLAB, “*fitgeotrans*” function). (2) Transform image from RGB to grayscale (“*rgb2gray*”). (3) Binarize image using fixed threshold of 100, followed by fill holes (“*imfill*”). (4) Remove object larger than 2000 pixels and smaller than 100 pixels (“*bwareaopen*”). (5) Display objects overlay.

### Data and Software Availability

#### Resources

The raw sequence data is deposited in the sequence read archive under accession SRP135960, available at https://www.ncbi.nlm.nih.gov/sra/SRP135960.

#### Software

The analysis software developed for this paper is available at https://github.com/linnarsson-lab, in repositories named cytograph and adolescent-mouse.

#### Additional Resources

We provide a companion wiki at http://mousebrain.org, with a report card for each cell type. The wiki can be browsed by taxon, cell type, tissue, and gene, with information on enriched genes, specific markers, anatomical location and more. The download section of the wiki makes available the following resources: (1) Aligned reads in the form of BAM files. (2) Quality-control results of each sample (10X Genomics Cell-Ranger QC output). (3) Expression data organized by individual Chromium sample, region, taxonomic group, and the entire final curated dataset. These files contain full metadata, graph layout, cluster assignments and cell type/state annotations, where appropriate.

Expression data is provided in Loom format (see http://loompy.org) and comes with an interactive, web-based viewer for explorative analysis. The wiki provides links to relevant Loom files, preloaded in the Loom viewer.

## Supplemental Information

Table S1. Samples, Related to Figure 1 A list of all Chromium samples used in the study, with metadata annotation including initial QC assessment.

Table S2. Comparison with Published, Validated Cell Types, Related to Figure 1 Table shows individual cell types (clusters) from published work in the cortex, striatum, sympathetic ganglia, spinal cord and dentate gyrus. For each reported cell type, the best match (by correlation of gene expression) in the current study is indicated, and the result of a manual assessment of the match is given. Open circle, perfect match; open dashed circle, near-perfect match; filled circle, mismatch.

Table S3. Cell Types and Metadata, Related to Figure 1 All the cell types (clusters) reported in this study are listed, ordered as in Figure 1C, along with annotation giving the cluster name, description, brain region, developmental compartment, putative neurotransmitter, taxonomy assignment, and probably location.

Table S4. Marker Genes, Related to Figure 1 For each cluster, a set of markers is given that uniquely identifies the cluster based on trinarization scores. Markers are read from left to right, and the cumulative specificity (difference in probabilty of identifying the cluster, relative to the second likeliest cluster), selectivity (number of clusters identified by the marker set), and robustness (probability of identifying the cluster) are indicated. A selectivity of 1 indicates a set of genes (reading left-to-right) that uniquely identifies the cluster among all clusters.

Table S5. Taxonomy, Related to Figure 1 A list of taxonomic units ordered hierarchically and corresponding to the dendrogram in Figure 1C. The number of cell types within each taxonomy level 4 unit is indicated on the right column.
